# Loss of Profilin3 Impairs Spermiogenesis by Affecting Acrosome Biogenesis, Autophagy, Manchette Development and Mitochondrial Organization

**DOI:** 10.3389/fcell.2021.749559

**Published:** 2021-11-04

**Authors:** Naila Umer, Lena Arévalo, Sharang Phadke, Keerthika Lohanadan, Gregor Kirfel, Dominik Sons, Denise Sofia, Walter Witke, Hubert Schorle

**Affiliations:** ^1^Department of Developmental Pathology, Institute of Pathology, University Hospital Bonn, Bonn, Germany; ^2^Institute for Cell Biology, University of Bonn, Bonn, Germany; ^3^Department of Membrane Biochemistry, Life and Medical Sciences (LIMES) Institute, University of Bonn, Bonn, Germany; ^4^Institute of Genetics, University of Bonn, Bonn, Germany

**Keywords:** profilin 3, acrosome biogenesis, sperm biology, globozoospermia, autophagy, male fertility

## Abstract

Profilins (PFNs) are key regulatory proteins for the actin polymerization in cells and are encoded in mouse and humans by four *Pfn* genes. PFNs are involved in cell mobility, cell growth, neurogenesis, and metastasis of tumor cells. The testes-specific PFN3 is localized in the acroplaxome–manchette complex of developing spermatozoa. We demonstrate that PFN3 further localizes in the Golgi complex and proacrosomal vesicles during spermiogenesis, suggesting a role in vesicle transport for acrosome formation. Using CRISPR/Cas9 genome editing, we generated mice deficient for *Pfn3*. *Pfn3^–/–^* males are subfertile, displaying a type II globozoospermia. We revealed that *Pfn3^–/–^* sperm display abnormal manchette development leading to an amorphous sperm head shape. Additionally, *Pfn3^–/–^* sperm showed reduced sperm motility resulting from flagellum deformities. We show that acrosome biogenesis is impaired starting from the Golgi phase, and mature sperm seems to suffer from a cytoplasm removal defect. An RNA-seq analysis revealed an upregulation of *Trim27* and downregulation of *Atg2a*. As a consequence, mTOR was activated and AMPK was suppressed, resulting in the inhibition of autophagy. This dysregulation of AMPK/mTOR affected the autophagic flux, which is hallmarked by LC3B accumulation and increased SQSTM1 protein levels. Autophagy is involved in proacrosomal vesicle fusion and transport to form the acrosome. We conclude that this disruption leads to the observed malformation of the acrosome. TRIM27 is associated with PFN3 as determined by co-immunoprecipitation from testis extracts. Further, actin-related protein ARPM1 was absent in the nuclear fraction of *Pfn3^–/–^* testes and sperm. This suggests that lack of PFN3 leads to destabilization of the PFN3–ARPM1 complex, resulting in the degradation of ARPM1. Interestingly, in the *Pfn3^–/–^* testes, we detected increased protein levels of essential actin regulatory proteins, cofilin-1 (CFL1), cofilin-2 (CFL2), and actin depolymerizing factor (ADF). Taken together, our results reveal the importance for PFN3 in male fertility and implicate this protein as a candidate for male factor infertility in humans.

## Introduction

Spermatogenesis is defined as the process of producing mature spermatozoa from spermatogonia. It comprises three phases, (i) spermatocytogenesis (mitotic), (ii) meiotic, and (iii) spermiogenesis. After a series of successive mitotic and meiotic divisions, spermiogenesis is the last step, where spermatozoa are reshaped to their final appearance. During spermiogenesis, haploid round spermatids are transformed into elongated spermatozoa (Oakberg, 1956; Agnew, 1997). This is achieved by a series of cellular reconstruction processes such as formation of the acrosome and tail, replacement of histones and protamine-induced DNA hypercondensation, removal of most of the cytoplasm, and rearrangement of mitochondria along the neck and tail region of the sperm. All mammalian spermatozoa are divided into two basic units based on their function—the head and the flagellum. Both units are shaped and assembled during spermiogenesis, which is the cytomorphogenic phase of spermatogenesis (Khawar et al., 2019). The acrosome is derived from the Golgi and is a secretory vesicle with an acidic pH, containing lysosomal hydrolases and other proteins such as acrosin and acrosin-binding proteins (Tang et al., 1982; Martínez-Menárguez et al., 1996). There are two main pathways that participate in acrosome formation, namely, (i) biosynthetic pathway—a route from trans-Golgi and (ii) endocytic pathway—a route from the endosome (Berruti and Paiardi, 2015). Acrosome biogenesis consists of four phases—(i) Golgi, (ii) cap, (iii) acrosomal, and (iv) maturation (Ramalho-Santos et al., 2001; Moreno and Alvarado, 2006). During the Golgi phase, proacrosomal granules fuse to form a single spherical acrosomal vesicle (Leblond and Clermont, 1952). In the cap phase, the acrosomal granule forms a head cap-like structure. Initially, there is transport of vesicles from Golgi to the acrosome. However, during the cap phase, the Golgi apparatus of the spermatid migrates to the front side of the cell, ending the transport of glycoproteins via the Golgi biosynthetic pathway (Tang et al., 1982; Ramalho-Santos et al., 2001). During the acrosomal phase, the head cap-like structure is elongated along the dorsal edge of the cell. In the final/maturation phase, the acrosome spreads over the anterior half in parallel to the elongation of the sperm head.

In spermatozoa, acrosomal abnormalities result in severe morphogenetic deformations leading to subfertility or infertility. In humans, this disorder is known as globozoospermia, affecting >0.1% of the infertile male population (Dam et al., 2007). Two types of globozoospermia have been described: type I globozoospermia, where all spermatozoa lack an acrosome and display a round head shape, and type II globozoospermia, where 20–60% spermatozoa lack an acrosome and display a round head shape (Bartoov et al., 1980). Acrosome formation is driven by proteins known to be involved in autophagy. There, the microtubule-associated protein LC3B plays an important role in autophagosome formation and is a widely used biomarker to detect autophagic flux. Further, autophagy-related genes such as *Atg7* (Wang et al., 2014), *Atg9* (Yefimova et al., 2019), and *Atg5* (Huang et al., 2020) are important for the acrosome biogenesis and male fertility. Autophagy itself is regulated, among others, by members of the TRIM protein family (Di Rienzo et al., 2020). In colorectal cancer cells, it has been shown that the overexpression of *Trim27* results in the activation of AKT signaling (Zhang et al., 2018). AKT is a serine threonine kinase, a downstream class of PI3K, and an activator of mTOR (Hahn-Windgassen et al., 2005). In turn, activated mTOR inhibits AMPK signaling, which leads to the suppression of autophagy (Liang et al., 2018).

The flagellum of the mammalian spermatozoa contains mitochondria, a fibrous sheath and central bundle of microtubules called axoneme (Lindemann and Lesich, 2016). The acroplaxome is a cytoskeletal scaffold present in the subacrosomal space within the spermatid. The function of the acroplaxome is the stabilization and anchoring of the acrosome during sperm nuclear elongation (Kierszenbaum and Tres, 2004). The manchette is a transient skirt-like structure present on the exterior pole of the developing spermatozoa. It is essential for the formation of a microtubular platform between the perinuclear ring surrounding the nucleus and the elongated sperm axoneme (Behnen et al., 2009). The exogenous clutching forces generated by F-actin hoops present in the apical region of the sperm nucleus coupled with endogenous modulating mechanism of the acrosome–acroplaxome–manchette complex are thought to play a role in the shaping of the spermatid head (Kierszenbaum and Tres, 2004). Spermiogenesis involves the extensive reshaping of the sperm head by the interaction of F-actin filaments with actin-interacting proteins such as profilins (PFNs) and cofilins (CFLs).

Two of the four known profilin gene family members *Pfn3* and *Pfn4* are expressed in the testes. Contrary to PFN1 and PFN2, PFN3 affected the kinetics of actin polymerization to a lesser extent, suggesting alternative and additional roles of PFN3. During the early steps of spermiogenesis, PFN3 is mainly observed in the acroplaxome of round spermatids and later detected in elongating spermatids at the acroplaxome–manchette complex (Behnen et al., 2009). However, the exact intracellular localization of PFN3 is not yet determined.

To understand the role of PFN3 in male fertility, we used CRISPR/Cas9 to generate *Pfn3*-deficient mice. Lack of *Pfn3* leads to male subfertility with sperm displaying impaired acrosome biogenesis due to defective transport of Golgi vesicles, malformed acrosomes, amorphous head shape, and manchette and flagellum deformities. These defects are reminiscent to type II globozoospermia. Using an RNA-seq analysis, we found *Trim27* upregulated in *Pfn3*-deficient mice. As a consequence, mTOR signaling was activated and AMPK was suppressed, leading to the accumulation of LC3B and SQSTM1 proteins, indicating a disturbance of the autophagic flux. Co-immunoprecipitation confirmed PFN3 interaction with TRIM27 from testis extracts. Further defects include loss of ARPM1 protein in the nuclear fraction of sperm and upregulation of actin-binding proteins ADF and CFL. Interestingly, in the mid-piece of the sperm flagellum, actin organization seemed not affected. These results suggest a surprising role of PFN3, an actin-related protein. Its main role seems to be the control of the *Trim27*-dependent signaling pathways, orchestrating acrosome formation, while the contribution to manchette formation seems rather minor.

## Materials and Methods

### Ethics Statement

Animal care, breeding setup, and experimental procedures were approved according to the German law of animal protection and in agreement with the approval of the local institutional animal care committees (Landesamt für Natur, Umwelt und Verbraucherschutz, North Rhine-Westphalia, approval ID: AZ84- 02.04.2013.A429).

### Designing CRISPR Guide RNA and Plasmid Construction

Guide RNAs (gRNAs) specific for *Pfn3* were designed using the online tool from Feng Zhang’s lab^1^ (Hsu et al., 2013). Designed gRNAs were annealed and cloned into pX330-U6-Chimeric_BB-CBh-hSpCas9 (plasmid # 42230 obtained from Addgene) (Cong et al., 2013) as described previously (Ran et al., 2013).

### Functionality of gRNAs Into mES Cells

The functionality of gRNAs was checked using E14Tg2a mES cells (kind gift of Christof Niehrs, IMB Mainz, Germany). On gelatinized cell culture dishes with standard ES cell medium without antibiotics, 3 × 10^5^ cells per well were seeded at 37°C and 7.5% CO_2_. After 3 h, cells were transfected with Lipofectamine 2000, pX330 containing gRNAs in a ratio of 1:3, according to the manufacturers’ protocol (Thermo Fisher Scientific, Waltham, United States). To remove DNA–Lipofectamine complexes, the medium was changed after 6–8 h to standard ES media.

### Generation of Pfn3 KO Mice

Superovulation was done by intraperitoneal injections of PMS (pregnant mare’s serum, 5 IU) and hCG (human chorionic gonadotropin, 5 IU) in C57BL/6J female mice and two females mated with one C57BL/6J male. At 0.5 dpc, zygotes from the oviducts were isolated and microinjection was done using an inverted microscope (Leica, Wetzlar, Germany) equipped with micromanipulators (Narishige, Japan) and piezo unit (Eppendorf, Hamburg, Germany). Injection pipettes (PIEZO 8-15-NS, Origio, Charlottesville, United States) were filled with Fluorinert (FC-770, Sigma-Aldrich, Taufkirchen, Germany) for appropriate piezo pulse propagation. Co-injection of Cas9 mRNA (100 ng/μl) (Sigma-Aldrich) and *in vitro*-transcribed single-guide RNAs (sgRNAs) (50 ng/μl each) were achieved into the cytoplasm of zygotes, as described previously (Yang et al., 2014). The zygotes that survived after microinjection were kept for 3 days in KSOM medium in a CO_2_ incubator. Resulting blastocysts were transferred into the uteri of pseudo-pregnant foster recipients. The alleles were registered with mouse genome informatics and received the following IDs: Pfn3^*em*1*Hsc*^ MGI:6384215, Pfn3^*em*2*Hsc*^ MGI:6384216, and Pfn3^*em*3*Hsc*^ MGI:6384217.

### Genotyping PCR

Genomic DNA was extracted from mice following the phenol/chloroform method and subjected for PCR. Gene-specific primers were used for PCR. PCR products were sequenced to identify the locus-specific deletions mediated by CRISPR/Cas9 genome editing. Primer list is given in the Supplementary Data (Supplementary Table 1).

### Fertility Analysis

For fertility analysis, total five–seven WT and KO male mice aged 10 to 12 weeks were individually housed with sexually mature WT C57BL/6J females in a controlled breeding experiment. Females were observed for the presence of vaginal plugs and pregnancies. The average litter size from pregnant females were calculated.

### Morphological Analysis

*Pfn3* knockouts were used for gross morphological analyses including body weight, testis weight, epididymis weight, and appearance of testes as compared to heterozygous and wild-type littermates (*n* = 13 animals/genotype).

### Epididymis Sperm Assessment

Mice epididymal sperm were extracted by multiple incisions of the cauda followed by a swim out for 30–60 min in M2 medium. Using a Neubauer hemocytometer, sperm count was determined (*n* = 13). A sperm vitality analysis was performed using eosin and nigrosine (E&N) staining, and sperm membrane integrity was accessed by hypo-osmotic swelling test (HOS test); 200 spermatozoa were calculated from three animals per genotype (WT, heterozygous, and knockouts) in a repetition, and results were expressed as percentage of live-to-dead sperm ratio. For all experiments, sexually mature males with an age of 2–6 months were used.

### Nuclear Morphology Analysis

For nuclear morphology analysis, after swim out, sperm cells were fixed and washed three times in 2% paraformaldehyde (PFA). After fixation, sample was diluted in a fixative and spread evenly on a glass slide and allowed to air dry. Slides were counterstained with DAPI (Carl Roth, Karlsruhe, Germany) as described previously (Skinner et al., 2019). Images were taken on the Leica DM5500 B/JVC KY-F75U digital camera. Images were analyzed using the ImageJ plugin “Nuclear morphology analysis v1.15.3” according to the developer’s instructions.

### Immunohistochemistry

The testes were fixed in Bouin’s solution for 4–24 h, washed with 70% ethanol to remove excess Bouin, embedded in paraffin, and sectioned at 5-μm thickness. Immunohistochemistry (IHC) was done in the Lab Vision PT module (Thermo Fisher Scientific) and Autostainer 480S (Medac, Hamburg, Germany) as published previously (Nettersheim et al., 2013). The primary antibodies against PFN3 (BEG6 kind gift of Prof. Dr. W. Witke), LC3B (ab58610, Abcam), p-mTOR (Cat#2971, Cell Signaling), RAB5 (PA-5-29022), and ARPM1 (27580-1-AP) were used.

### Protein Extraction and Western Blot Analysis

For the crude protein extracts, the testes were homogenized into 500–900 μl RIPA buffer (Thermo Fisher Scientific, Waltham, United States) using a Dounce homogenizer (Sartorius, Göttingen, Germany). The homogenized mixture was kept on ice for 15 min followed by 15 min of centrifugation at 4°C and 13,000 rpm. In order to load equal amounts of protein, supernatant was used to measure the protein concentration using the Pierce BCA Protein Assay Kit (Thermo Fisher Scientific, Waltham, United States). Cytoplasmic and nuclear fractions were separated as described previously (Hara et al., 2008). SDS gel followed by western blot was performed as described previously (Nettersheim et al., 2016) with primary antibodies against PFN3 (BEG6 kind gift of Prof. Dr. W. Witke), CFL1, CFL2, and ADF (kind gift of Prof. Dr. W. Witke), and ARPM1 (27580-1-AP, ProteinTech), ATG2A (Cat#PA5-77794, Thermo Fisher Scientific), TRIM27 (12205-1-AP), LC3B (ab58610, Abcam), SQSTM1 (Cat#5114, Cell Signaling), mTOR (Cat#2972, Cell Signaling), p-mTOR (Cat#2971, Cell Signaling), and AMPK (Cat#5831, Cell Signaling) were used.

### Immunoprecipitation

Protein immunoprecipitation (IP) was performed on whole adult testis lysate. Testis tissue was homogenized in RIPA buffer followed by sonication at high speed for five cycles of 30-s ON/30-s OFF with a Bioruptor^®^ sonication device, and protein extraction was done as described above. Co-IP was performed using Dynabeads^®^ M-280 Sheep Anti-Rabbit IgG as described by the manufacturer’s protocol. The captured and eluted proteins were separated by SDS-PAGE and transferred to nitrocellulose membranes in preparation for immunoblot analysis.

### cDNA Synthesis and Real-Time PCR

RNA was extracted from testes tissue after removal of the tunica albuginea using TRIzol reagent according to manufacturer’s protocol (Life Technologies, Carlsbad, United States). The concentration and purity of isolated RNA was measured by a NanoDrop instrument (Peqlab, Erlangen, Germany). After DNAseI treatment of RNA, cDNA was synthesized using RevertAid First Strand cDNA Synthesis Kit (Fermentas, St. Leon-Rot, Germany). Quantitative reverse transcription polymerase chain reaction (qRT-PCR) was performed on ViiA 7 Real-Time PCR System (Applied Biosystems, distributed by Life Technologies) using Maxima SYBR Green qPCR Master Mix (Life Technologies) as described previously (Jostes et al., 2017). At the end of each PCR run, a melting point analysis was performed. GAPDH was used as reference gene for data normalization.

### RNA-Seq Analysis

RNA integrity (RIN) was determined by the sequencing facility (UKB sequencing core facility) using the RNA Nano 6000 Assay Kit with the Agilent Bioanalyzer 2100 system (Agilent Technologies, Santa Clara, CA, United States). RIN values ranged from 8.2 to 10 for all samples. RNA sample quality control and library preparation were performed by the sequencing facility (UKB sequencing core facility), using the QuantSeq 3’-mRNA Library Prep (Lexogen). RNA-seq was performed on the Illumina HiSeq 2500 V4 platform, producing > 10 million, 50-bp 3’-end reads per sample.

All samples were mapped to the mouse genome (GRCm38.89). Mapping was done using HISAT2 2.1 (Kim et al., 2015). Transcript quantification and annotation was done using StringTie 1.3.3 (Pertea et al., 2015). Gene annotation information for the mouse genome was retrieved from the Ensembl FTP server^2^ (GRCm38.89). We used the python script (preDE.py) included in the StringTie package to prepare gene-level count matrices for analysis of differential gene expression.

Differential expression was tested with DESeq2 1.16.1 (Love et al., 2014). Pseudogenes were removed from the count matrices based on “biotype” annotation information extracted from Biomart (R-package biomaRt) (Durinck et al., 2005). Low counts were removed by the independent filtering process implemented in DESeq2 (Bourgon et al., 2010). The adjusted *p*-value (Benjamini–Hochberg method) cutoff for DE was set at <0.05, and log2 fold change of expression (LFC) cutoff was set at >1.5.

The datasets presented in this study can be found in online repositories. The names of the repository/repositories and accession number(s) can be found at: https://www.ncbi.nlm.nih.gov/geo/, GSE171068.

### Transmission Electron Microscopy

Epididymal sperm and testis tissue were washed in phosphate buffered saline (PBS) and fixed in 1.5% glutaraldehyde in 0.1 M cacodylate buffer (pH 7.4). Sperm cells were rinsed in 0.1 M cacodylate buffer and fixed in 2% osmium tetroxide again followed by an additional washing in 0.1 M cacodylate buffer. Afterward, dehydration was performed by an increasing ethanol concentration, terminated by two incubations in propylene oxide for 15 min, and an interim staining in 0.5% uranyl acetate. Samples were stored in propylene oxide and EPON mixture (1:1) overnight and followed by embedding in EPON for 24 h at 70°C. Ultrathin sections were picked up on grids, stained with 3.5% uranyl acetate for 25 min and lead citrate solution for 7 min, and images were taken on the Philips CM 10 TEM. For immunogold labeling, prior to fixation in glutaraldehyde, sections were incubated with PFN3 antibody (1:50) for 1 h. Followed by washing, gold-conjugated anti-rabbit IgG (10 nm) secondary antibody (1:50) was incubated for 1 h. In the negative control, primary antibody was omitted. Finally, the sections were post-stained with uranyl acetate.

### Scanning Electron Microscopy

Sperm cells were fixed in primary fixative for 30 min at 4°C: 1% glutaraldehyde and 0.4% formaldehyde in 0.1 M sodium cacodylate buffer (pH 7.2). After washing the samples three times in 0.1 M sodium cacodylate buffer for 5 min, they were post-fixed with 1% OsO_4_ in 0.1 M sodium cacodylate buffer for 30 min followed by three washing steps. Images were taken using a Verios 460L (FEI, Eindhoven, Netherlands) (Nussdorfer et al., 2018) equipped with a STEM 3 detector.

### Immunofluorescence Staining

Immunofluorescence staining was performed on testis sections using peanut agglutinin (PNA)- fluorescein isothiocyanate (FITC) Alexa Fluor 488 conjugate (Molecular Probes, Invitrogen), anti-mouse GM130 (610823, BD Biosciences, United States), and anti-rabbit TGN46 (JF1-024, Thermo Fischer Scientific) to check the acrosome biogenesis and *cis-* and *trans-*Golgi structural organization, respectively; 5-μm testis tissue sections were taken on slides. Deparaffinization on Bouin’s fixed paraffin-embedded testis tissue was performed by immersing them in xylol for two times, followed by dehydration steps in 100, 95, and 70% alcohol. After washing with PBS twice, tissue sample was permeabilized in 0.1% Triton X-100 for 5 min at 37°C, followed by blocking in 1% bovine serum albumin (BSA) for 1 h at room temperature. Tissue sections were incubated with diluted PNA-FITC in PBS at room temperature for 30 min; next, slides were washed in PBS for two times. Tissue sections were incubated with GM130 and TGN46 antibodies diluted at 2 μg/ml in 0.1% BSA at 4°C for overnight, followed by PBS washing, and respective secondary antibodies were used for 1 h at room temperature. Tissue sections were mounted with ROTI^®^Mount FluorCare DAPI (Carl Roth, Karlsruhe, Germany). Images were taken within 24 h using an LSM 710 (Zeiss, Oberkochen, Germany).

For sperm immunofluorescence, after swim out, sperms were washed two–three times in PBS followed by fixation in 4% paraformaldehyde for 20 min at room temperature. After washing with PBS, spermatozoa were incubated with PNA-FITC and Mito Red (5 nM, 53271; Sigma Aldrich) diluted in PBS at room temperature for 30 min. For manchette staining, germ cell population was isolated from testes as described previously (Li W. et al., 2015), and staining with anti-alpha tubulin antibody (clone DM1A Alexa Flour 488 conjugate; 16-232 Sigma Aldrich) was performed as described previously (Rotgers et al., 2015). Next, spermatozoa were plated onto Superfrost Plus Microscope Slides (Thermo Fisher Scientific), mounted with ROTI^®^Mount FluorCare DAPI (Carl Roth, Karlsruhe, Germany). Images were taken within 24 h using an LSM 710 (Zeiss, Oberkochen, Germany).

### STED Imaging

Epididymal sperm cells after swim out were diluted into PBS (1:10); 400 μl of diluted sperm were loaded on poly-L-lysine (CAS 25988-63-0, Sigma Aldrich)-coated coverslips in a six-well plate and air dried for 30 min at 37°C. After removing the PBS, sperm cells were fixed in 4% PFA followed by quenching with 50 mM NH_4_Cl for 15 min. Sperm cells were permeabilized by 0.02% Triton X-100 for 3 min followed by washing with PBS, incubation for 1 h at RT with Phalloidin ATTO647 (1/1,000 in 3% BSA, ab176759, Abcam), and mounting with ProLong Gold Antifade (#P36930, Life Technologies). STED micrographs were acquired using a four-channel easy3D super-resolution STED optics module (Abberior Instruments, Göttingen, Germany) coupled with an Olympus IX73 confocal microscope (Olympus, Tokyo, Japan) and equipped with an UPlanSApo × 100 (1.4 NA) objective (Olympus, Tokyo, Japan) (Mikuličić et al., 2019), available in the LIMES Imaging Facility.

### Sperm Motility Analysis

Epididymal sperm were incubated in TYH medium at 37°C for 20 min, after swim out sperm cells were diluted into the TYH medium supplemented with BSA. The diluted cell suspension was placed onto the pre-warmed (37°C) slide, and video was recorded using the Basler Microscopy ace camera (acA 1920-155uc) at 300 frames, streaming video using pylon Viewer (v.5.0.11.10913, Basler AG, Ahrensburg, Germany). Sperm motility was evaluated by using the OpenCASA program as described previously (Alquézar-Baeta et al., 2019).

### Acrosomal Reaction

Sperm were isolated from cauda epididymis and allowed to swim out in M2 medium for 10 min. Capacitation was induced by incubating in HTF medium for 90 min at 37°C, 5% CO_2_. To induce acrosome reaction, calcium ionophore A23187 (10 μM, c7522; Sigma Aldrich) was added. After 15 min, sperm were spread on glass slides, air dried, and fixed in methanol for 3 min at RT. Followed by PBS washing, sperm were subjected to Coomassie brilliant blue staining (2% *w*/*v* G250) for 3 min as described previously (Liu et al., 2019). Followed by PBS washing two times for 2–3 min each, mounting was performed by using ROTI^®^Mount FluorCare (HP 21.1 Carl Roth). Two hundred spermatozoa for each genotype (*n* = 3) were assessed by a bright field microscope.

### Statistics

The mean of all values for a particular data set has been represented in graphical form. Error bars have been used to denote standard deviation. Student’s *T*-test (two-tailed unpaired and one-tailed paired) and ANOVA (Tukey’s *post hoc*) were performed to ascertain the significance of the variability in data. *P*-value less than 0.05 (*p* < 0.05^∗^, <0.005^∗∗^, 0.001^∗∗∗^) was considered as statistically significant.

## Results

### Ultrastructural Analysis of PFN3 Localization in Mouse Testes

**FIGURE 1:**
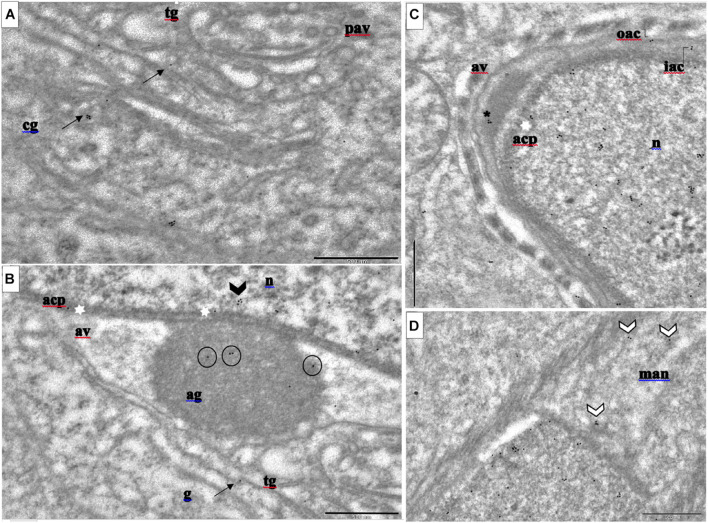
Immunogold labeling of PFN3 in developing mouse spermatid during acrosome biogenesis. **(A)** *cis*- and *trans-*Golgi network; immunogold particles in cg and tg = black arrows. **(B)** Golgi phase; immunogold labeling highlighted in ag = circles, acp = white stars, and n = arrow head. **(C)** Cap phase; immunogold particles highlighted in av = asterisk. **(D)** Man immunogold labeling = white arrow heads. cg, cis-Golgi; tg, trans-Golgi; ag, acrosomal granule; n, nucleus; acp, acroplaxome; av, acrosomal vesicle. Scale bar = 500 nm. **(A,B)** are from the same image (ref.to Supplementary Figure 2A).

We examined the distribution of PFN3 at ultrastructural level during spermiogenesis with a PFN3 polyclonal antibody and a gold-conjugated secondary antibody using immunoelectron microscopy. With immunogold labeling, we were able to determine localization of PFN3 in sub-domains of cellular compartments at the *cis*- and *trans-* Golgi network, the acroplaxome, mitochondria, and the manchette. In the Golgi phase, the electron dense giant acrosomal granule forms from the numerous Golgi-derived proacrosomal vesicles (Supplementary Figure 1A). Gold particles were detected representing PFN3 in the *cis-* and *trans-*part of the Golgi network adjacent to the nuclear pole of the round spermatid (Figure 1A and Supplementary Figures 1B,C,E,F, arrows). The *cis*-part is responsible for organizing and sorting of proteins imported from the endoplasmic reticulum (ER) and transported to the *trans-*part of the Golgi network where they are modified and exported as proacrosomal vesicles (Supplementary Figure 1E). Immunogold labelling was detected in the acrosomal granule (Figure 1B, circle), a giant structure attached to the middle of the acroplaxome (Figure 1B, white stars) of developing spermatozoa. We detected gold traces in the mitochondria, which are responsible for providing energy for flagellum propelling and germ cell differentiation (Supplementary Figures 1D,E, white arrows). Some of the gold particles were detected in the inner membrane of acrosome–acroplaxome interface, suggesting that PFN3 contributes to the attachment of acrosomal granule (Supplementary Figure 1E, white stars). In the cap phase (Supplementary Figure 1G), we detected PFN3 in the acrosomal vesicles (Figure 1C and Supplementary Figures 1H,I, asterisk), which are responsible for the formation of acrosome. In addition, we detected few gold traces in the nucleus (Figures 1B,C and Supplementary Figures 1H–M), suggesting that PFN3 might play a role in sperm nuclear shaping. Immunogold labelling also showed PFN3 localization in inner and outer membranes at the leading edge of the acrosome (Supplementary Figures 1H,I,K, elbow). Gold traces were also detected in highly specialized structures of elongating spermatozoa such as the manchette (Figure 1D and Supplementary Figures 1J,M, white arrow heads), the acroplaxome marginal ring (Supplementary Figure 1O, double arrows), as well as flagellum formation (Supplementary Figures 1N,Q,T). Cross sections of the flagellum showed gold traces in the mitochondria, which are gathered around the axoneme (Supplementary Figures 1U–W) to form the mitochondrial sheath in the sperm mid-piece (Supplementary Figure 1W). So, PFN3 is detected in the Golgi sub-domains, the acroplaxome–manchette complex, and the mitochondria, suggesting a role for PFN3 in acrosome biogenesis, sperm head shaping, and tail formation. The negative control is given in the Supplementary Data (Supplementary Figures 1Y,Z).

### Pfn3-Deficient Male Mice Display Sub-Fertility and Low Sperm Quantity and Quality

**FIGURE 2:**
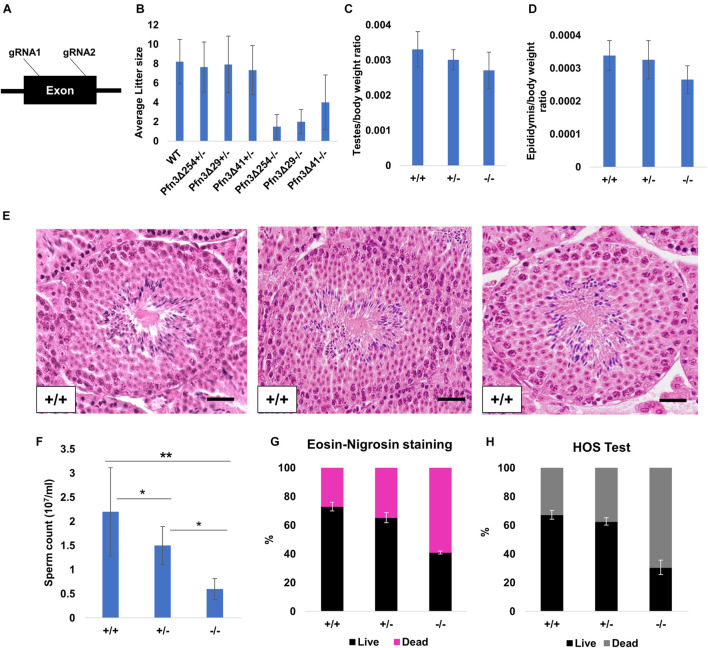
Generation and characterization of *Pfn3*-deficient mice. **(A)** Schematic representation of *Pfn3* genomic locus with targeting sites of designed gRNAs. **(B)** Mating statistics of wild-type, *Pfn3*Δ254, *Pfn3*Δ41, and *Pfn3*Δ29 heterozygous and homozygous (*n* = 5/*Pfn3*-deficient mouse model) males. Successful mating of heterozygous and homozygous males with wild-type females was indicated by the presence of a vaginal plug at 0.5 dpc. **(C,D)** Relative weights of testes and cauda epididymis are comparable between all three genotypes of *Pfn3*Δ254 (*n* = 13). **(E)** H & E staining on *Pfn3^+/+^*, *Pfn3^+/–^*, and *Pfn3^–/–^* testes section. **(F)** Sperm count comparison in *Pfn3^+/+^*, *Pfn3^+/–^*, and *Pfn3^–/–^* littermates (*n* = 13). **(G)** Eosin and nigrosine staining on biological replicates (*n* = 3) of *Pfn3^+/+^*, *Pfn3^+/–^*, and *Pfn3^–/–^* sperm. **(H)** Hypo-osmotic swelling test on biological replicates (*n* = 3) of *Pfn3^+/+^*, *Pfn3^+/–^*, and *Pfn3*^–/–^ sperm. At least 200 spermatozoa were evaluated per sample.

We generated *Pfn3*-deficient mice by injecting Cas9 mRNA and two sgRNAs into the cytoplasm of fertilized eggs targeting the exon of the gene (Figure 2A). Six pups carrying CRISPR/Cas9-induced mutations were identified (Supplementary Figure 2A) and three mouse lines, *Pfn3*Δ254, *Pfn3*Δ41, and *Pfn3*Δ29 harboring deletions of 254, 41, and 29 bp, respectively, were established by backcrossing with C57BL/6 mice. All deletions result in null alleles since they encode for frame shifts in the PFN3 reading frame leading to premature translational termination (Supplementary Figure 2C). qRT-PCR, western blotting, and IHC confirmed the deletion of *Pfn3* in the *Pfn3*-deficient mice (Supplementary Figure 2D). Western blotting displayed a signal in testis/post-natal testis (day 28). All other tissues tested were negative for PFN3. Similarly, IHC produced a signal for PFN3 in the testis but not in the brain and kidney sections. These results indicate the specificity of the PFN3 antibody (Supplementary Figure 2E). IHC of WT testis section using anti-PFN3 antibody showed a strong expression of PFN3 in the nuclear region of elongated spermatids (Supplementary Figure 2D). In addition, IF using PFN3 antibody on purified germ cells revealed that PFN3 is localized to the cytoplasm of round spermatids. As round spermatids develop into elongating spermatids, PFN3 is localized in a punctuated manner around the acrosomal region (Supplementary Figure 2F). In non-acrosome-reacted spermatozoa, PFN3 is located in the acrosome and the nucleus. In acrosome-reacted spermatozoa, PFN3 can be detected in the head region (Supplementary Figure 2G). Males heterozygous for either *Pfn3*Δ254, *Pfn3*Δ41, and *Pfn3*Δ29 produced an average litter size of 7.6, 7.9, and 7.3, respectively (Figure 2B), which is comparable to wild-type mice with a mean litter size of 8.2 (Biggers et al., 1962). Homozygous *Pfn3* male mice are sub-fertile since they produced an average litter size of 1.5, 2, and 4 for *Pfn3*Δ254, *Pfn3*Δ41, and *Pfn3*Δ29, respectively (Figure 2B).

Adult *Pfn3*-deficient males for either *Pfn3*Δ254, *Pfn3*Δ41, or *Pfn3*Δ29 mated with females normally as vaginal plugs were clearly detectable. Relative weights of the testes and epididymis of *Pfn3*-deficient mice (*Pfn3*Δ254) were slightly reduced; however, that reduction was not significant (Figures 2C,D). Histological analysis using hematoxylin and eosin H & E) staining on *Pfn3^+/+^, Pfn3^+/–^*, and *Pfn3^–/–^* testes sections showed normal morphology of seminiferous tubules (Figure 2E). This suggests that deletion of *Pfn3* left the gross morphology of the testes and epididymis unaffected.

However, *Pfn3^+/–^* and *Pfn3^–/–^* males showed a significant reduction in sperm count (Figure 2F). Next, E&N staining was performed to assess the vitality of spermatozoa. E&N distinguish live (whitish in color, arrow head) from dead sperm (pink in color, arrow in Supplementary Figure 2K). A percentage of live sperm in the range of 60–80% is considered normal, borderline 40–60%, and below 40% is considered abnormal. E&N staining showed that in heterozygous males, the percentage of viable sperm is in the normal range, while in case of *Pfn3*-deficient mice, the percentage of viable sperm was at borderline (∼40%) (Figure 2G). Further, the HOS test was performed to check the integrity of sperm membrane, where intact (live) sperm displays a swelling of the tail (Supplementary Figure 2M). The percentage of hypo-osmotic reactive sperm for *Pfn3^–/–^* mice was again at borderline (Figure 2H). Male mice for *Pfn3*Δ41 and *Pfn3*Δ29 have the same phenotype as *Pfn3*Δ254 mice (Supplementary Figures 2H–N). In conclusion, loss of *Pfn3* impinges not only on sperm quantity but also sperm quality.

### Sperm Head Morphology Is Altered in Pfn3-Deficient Mice

**FIGURE 3:**
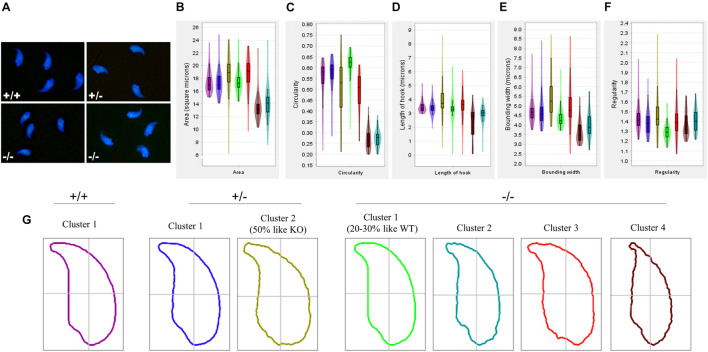
Amorphous nuclear morphology of sperm cells in *Pfn3*-deficient mice. **(A)** Sperm cells stained with DAPI. **(B)** Area, **(C)** circularity, **(D)** length of hook, **(E)** bounding width, and **(F)** regularity of *Pfn3^+/+^*, *Pfn3^+/–^*, and *Pfn3^–/–^* sperm cells. **(G)** Head shape of *Pfn3^+/+^* sperm cells, two clusters for *Pfn3^+/–^* sperm cells, and four clusters for *Pfn3^–/–^* sperm cells.

We next used geometric morphometric analysis to analyze sperm head shape in detail. DAPI-stained sperm cells showed altered head shape for *Pfn3^–/–^* compared to control (Figure 3A). An analysis of 994 nuclei revealed (334 *Pfn3*^+/+^, 289 *Pfn3^+/–^*, and 371 *Pfn3^–/–^*) that *Pfn3*-deficient sperm shows alterations in area (Figure 3B), circularity (Figure 3C), length of hook (Figure 3D), bounding width (Figure 3E), and regularity (Figure 3F). Next, clustering was performed to categorize the sperm heads. Of note, 50% of heterozygous sperm nuclei are similar to WT, while 50% have abnormal morphology similar to *Pfn3*-deficient sperm nuclei (Figure 3G). This finding suggests a gene–dosage effect, which, however, does not seem to affect the mating success of *Pfn3^+/–^* males. Interestingly, 70–80% of *Pfn3*-deficient sperms showed irregular/round head morphology. Our analysis clearly shows that PFN3 plays a role in shaping of the sperm head during spermiogenesis.

### Sperm Motility Is Reduced in Pfn3-Deficient Mice

To analyze the swimming properties of *Pfn3*-deficient sperm, we performed computer-assisted semen analysis (CASA). Compared to WT and Het sperm samples (Table 1) *Pfn3^–/–^* sperm showed significantly reduced progressive and total motility. The other sperm motility parameters such as curvilinear velocity (VCL), straight-line velocity (VSL), and average path velocity (VAP) are reduced, but the difference is not significant when compared to sperm samples of controls. Of note, the observed reduction in sperm motility most likely is the reason of the decreased sperm count in *Pfn3*-deficient mice.

**TABLE 1:** Motility parameters were analyzed for *Pfn3^+/+^*, *Pfn3^+/–^*, and *Pfn3^–/–^* sperms using OpenCASA software (*n* = 3/genotype).

	VSL mean (μm/s)	VCL mean (μm/s)	VAP mean (μm/s)	Progressive motility (%)	Motility (%)
*Pfn3* ^+/+^	62.33 ± 1.2	218.5 ± 11.5	122.3 ± 4.7	47.2 ± 1.7	59 ± 5.9
*Pfn3* ^+/–^	58.46 ± 1.9	187 ± 5	107 ± 5.2	45.65 ± 9.9	52.11 ± 7.8
*Pfn3* ^–/–^	49.57 ± 13.8	170.857 ± 49.14	91.39 ± 25.03	32.388 ± 7.4*	34.91 ± 7.9**

*Data is presented as mean ± SD using ANOVA (Tukey’s post hoc test). VSL, straight-line velocity (μm/s); VCL, curvilinear velocity (μm/s); VAP, average path velocity (μm/s).*

### Impaired Acrosome Biogenesis in Pfn3-Deficient Mice

**FIGURE 4:**
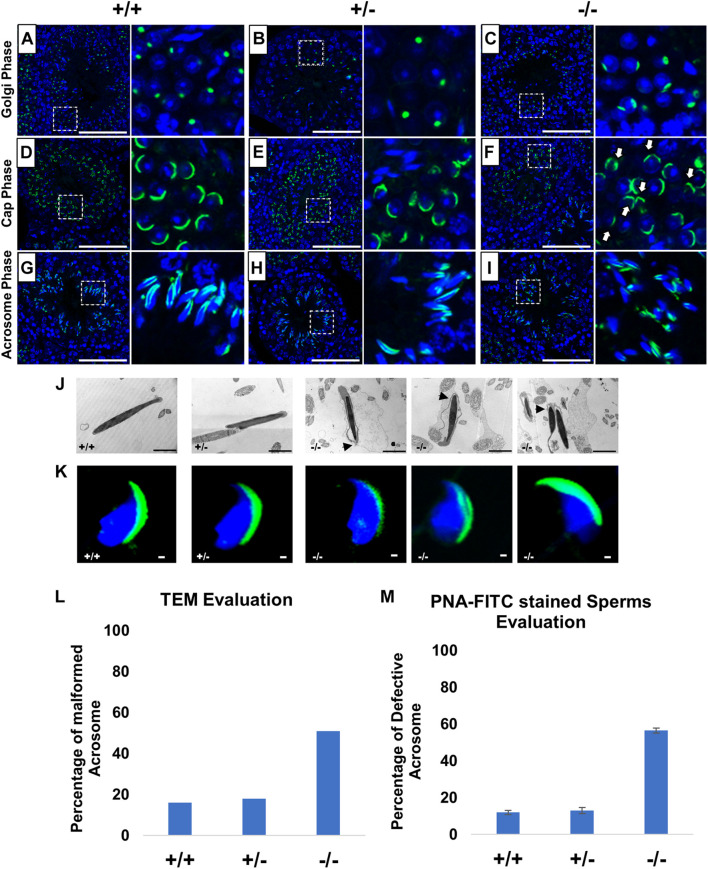
Impaired acrosome biogenesis in *Pfn3*-deficient mice. Adult testis sections of *Pfn3^+/+^* (left panel), *Pfn3^+/–^* (middle panel), and *Pfn3*-deficient (right panel) mice; the developing acrosome was labeled with PNA-FITC (green) and cell nuclei were stained with DAPI (blue); inset panel showed the Golgi/cap/acrosomal phase of the tubules displayed in the main panel (*n* = 3). In the Golgi phase, proacrosomal granule (green) labeled by PNA-FITC for **(A)** *Pfn3^+/+^*, **(B)** *Pfn3^+/–^*, and **(C)** *Pfn3^–/–^* round spermatozoa. In the cap phase, acrosomal caps (green) stained for **(D)** *Pfn3^+/+^*, **(E)** *Pfn3^+/–^*, and **(F)** *Pfn3^–/–^* round spermatozoa (white arrows show fragmented cap structures). In the acrosome phase, PNA-FITC-labeled acrosomal area on **(G)** *Pfn3^+/+^*, **(H)** *Pfn3^+/–^*, and **(I)** *Pfn3^–/–^* elongated spermatids. Scale bar = 20 μm. **(J)** Ultrastructure analysis using TEM revealed the acrosomal structures of *Pfn3^+/+^*, *Pfn3^+/–^*, and *Pfn3^–/–^* sperm cells. Scale bar = 2 μm. **(K)** Immunofluorescence staining using PNA-FITC (green) on epididymal sperm cells of *Pfn3^+/+^*, *Pfn3^+/–^*, and *Pfn3^–/–^* mice (*n* = 3). Scale bar = 20 μm. **(L)** TEM evaluation shows the percentage of malformed acrosomes in *Pfn3^+/+^*, *Pfn3^+/–^*, and *Pfn3^–/–^* sperm cells (*n* = 2). **(M)** A graph represents the PNA-stained defective acrosome percentage of *Pfn3^+/+^*, *Pfn3^+/–^*, and *Pfn3^–/–^* sperm cells (*n* = 3). Two hundred spermatozoa were counted per genotype.

PFN3 is detected in actin-rich structures such as acrosome–acroplaxome and Golgi complex, suggesting a role in acrosome biogenesis. In order to investigate the development of acrosome, we used PNA-FITC fluorescence labeling on testes sections. In tubules of wild-type (Figure 4A) and heterozygous males (Figure 4B), Golgi-phase spermatids showed developing acrosomes forming a homogenous single cluster on the apical face of cell nuclei. In mutant spermatids, PNA staining was scattered, suggesting a less uniform acrosomal compartment (Figure 4C). This abnormal formation of the acrosome was further observed in the next step, the cap phase. Here, the acrosome forms a cap-like structure covering the anterior half on round spermatids as seen in WT (Figure 4D) and heterozygous (Figure 4E) sections. In *Pfn3^–/–^*, the cap structures were more unevenly distributed in round spermatids (Figure 4F). In the acrosomal phase, as spermatids started to elongate during sperm head remodeling, these defects were more prominent, and the acrosomal content failed to form an arrow-like shape in elongating spermatids of *Pfn3^–/–^* mice (Figure 4I), compared to WT (Figure 4G) and heterozygous (Figure 4H). These results indicate that loss of PFN3 impairs acrosomal biogenesis already at the Golgi phase.

To understand the impaired acrosome biogenesis more in detail, an ultrastructure analysis using transmission electron microscopy (TEM) was performed on developing spermatids. In the Golgi phase, proacrosomal granules originate from the *trans*-Golgi and fuse to form a single, large acrosome vesicle attaching itself in the middle of the acroplaxome on the nuclear surface as seen in WT (Supplementary Figure 3A) and heterozygous cells (Supplementary Figure 3B). However, sections of *Pfn3*-deficient testes showed that the proacrosomal vesicle fail to attach in the middle of the acroplaxome and did not form a dense giant vesicle (Supplementary Figure 3C). In the cap phase, the proacrosomal granule starts to develop and flattens over the nucleus, forming the acrosomal cap, displayed in WT (Supplementary Figure 3D) and heterozygous spermatids (Supplementary Figure 3E). However, in *Pfn3*-deficient testes, the acrosomal vesicle fails to form a continuous cap-like structure and present as a detached granule from the acroplaxome (Supplementary Figure 3F). In the acrosome phase, the cap continues to develop as an arrow-like cover spanning the anterior two-third of the nucleus as indicated in WT (Supplementary Figure 3G) and heterozygous spermatids (Supplementary Figure 3H). Finally, at the end of maturation phase, acrosome formation is completed (Supplementary Figures 3I,J). In *Pfn3*-deficient sperm, during the acrosomal and maturation phases, the acrosomal granule fails to develop further (Supplementary Figures 3K,L).

In order to see the acrosomal defect in mature sperm, we next analyzed epididymal sperm cells of *Pfn3*-deficient males using TEM and immunofluorescence staining (PNA-FITC). An ultrastructural analysis and acrosomal labelling revealed malformation of the acrosomal region in epididymal sperms of *Pfn3^–/–^* mice. Sperm cells present with elongating projections (highlighted by arrows), detached acrosome, abnormal acrosomal covering, and impaired removal of cytoplasm (Figure 4J). Similarly, mature sperm stained with PNA showed malformation of the acrosome in addition to abnormal sperm head morphology (Figure 4K). Imaging of 200 spermatozoa revealed that, in *Pfn3^–/–^* male mice, 51–56% of spermatozoa display malformed acrosome compared to heterozygous (18%) and WT (16%) spermatozoa (Figures 4L,M).

### Significant Decrease of Acrosome-Reacted Sperm in Pfn3-Deficient Mice

**FIGURE 5:**
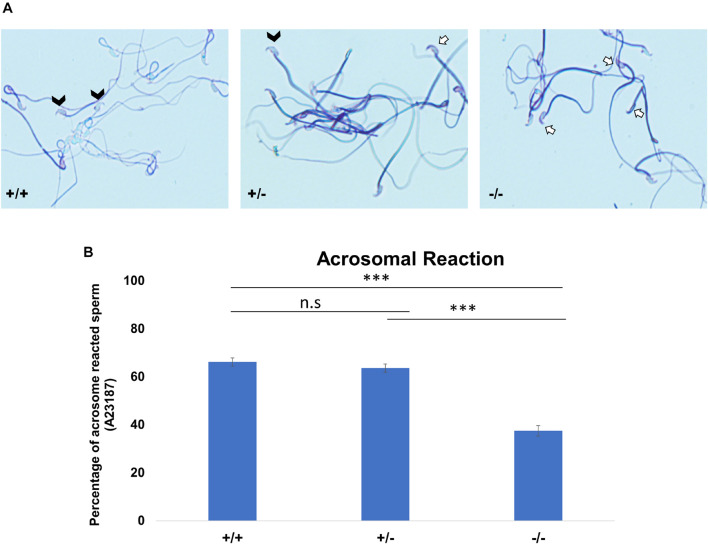
The acrosomal reaction (AR) using calcium ionophore. **(A)** Coomassie-stained sperm cells of *Pfn3^+/+^*, *Pfn3^+/–^*, and *Pfn3^–/–^*. Black arrow heads indicate successful acrosomal reaction took place. A white arrow indicates a crescent-shape acrosome on sperm head, indicating acrosomal reaction did not take place. **(B)** Percentage of acrosomal-reacted sperm for *Pfn3^+/+^*, *Pfn3^+/–^*, and *Pfn3^–/–^* (*n* = 3 biological replicates/genotype, ^∗∗∗^*p* < 0.0005, Student’s *t*-test, one tail, paired).

As a consequence of the malformed acrosome, we reasoned that the acrosomal reaction (AR) could be impaired. We used the A23187 to induce AR. Acrosomal status was evaluated using Coomassie staining; intact acrosomes were stained dark blue as a crescent-like shape on the top of the sperm head (Figure 5A, white arrows), whereas acrosome-reacted sperm showed that the crescent-like shape (acrosome) on the top of the sperm head was not present (Figure 5A, white arrow heads). The rate of A23187-induced AR was significantly reduced in *Pfn3*-deficient sperm. Upon exposure to A23187, more than 60% of sperm from *Pfn3*^+/+^ mice and ∼60% of sperm from *Pfn3^+/–^* mice (Figure 5B) underwent acrosome exocytosis, whereas the AR occurred only in 37.5% of sperm from *Pfn3^–/–^* mice (Figure 5B). This result indicates that the observed acrosome malformation impairs the acrosome exocytosis.

### Disrupted Golgi Network in Pfn3-Deficient Mice

**FIGURE 6:**
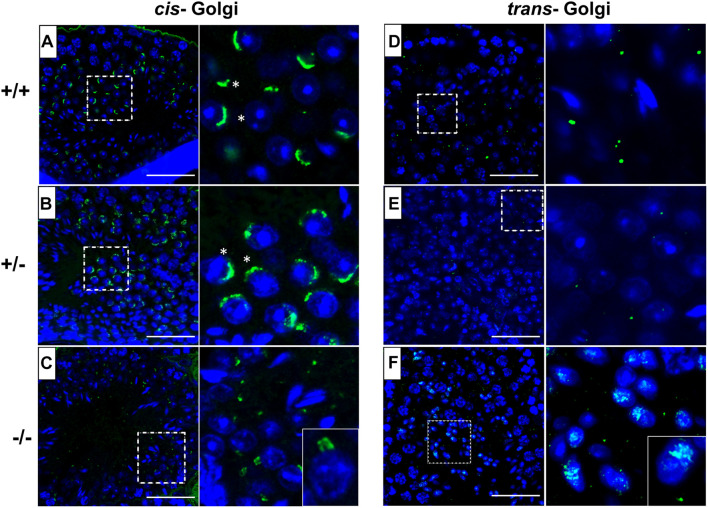
*Pfn3^+/+^*, *Pfn3^+/–^*, and *Pfn3^–/–^* testis sections stained for the *cis-* (GM130 antibody) and *trans-* (TGN46 antibody) Golgi compartment (green) and nuclei (DAPI, blue) (*n=3*). **(A)** WT and **(B)** heterozygous, and **(C)** *Pfn3^–/–^* spermatozoa stained for *cis*-Golgi compartment (asterisks). **(D)** WT, **(E)** heterozygous, and **(F)** *Pfn3^–/–^* spermatozoa stained for *trans-*Golgi network. Scale bar = 50 μm.

In order to investigate the underlying mechanism of impaired acrosome biogenesis in *Pfn3*-deficient mice, we performed immunofluorescence (IF) staining by using GM130 and TGN46 antibodies, markers for *cis-* and *trans-*Golgi network, respectively. GM130 plays a crucial role in vesicle tethering, fusion, and maintaining *cis*-Golgi structural integrity (Tiwari et al., 2019), while TGN46 is important for formation of exocytic vesicles and secretion from the *trans-*part of the Golgi network (Huang et al., 2019). IF revealed *cis-* and *trans-*Golgi predominantly concentrated at one pole of the *Pfn3*^+/+^ (Figures 6A,D) and *Pfn3^+/–^* (Figures 6B,E) spermatids, whereas the *Pfn3^–/–^* spermatids showed defects and disorganization in *cis-* (Figure 6C) and *trans*-Golgi network (Figure 6F). These results indicate that loss of *Pfn3* leads to disruption of the Golgi sub-domains causing defects in Golgi-derived proacrosomal vesicles leading to acrosome malformations.

Berruti and Paiardi (2015) published that in addition to the Golgi-derived biosynthetic pathway, the endocytic pathway contributes to acrosome biogenesis. In order to check whether loss of PFN3 affects the endocytic pathway, we performed IHC staining using anti-Rab5 antibody on testis sections for all three genotypes. Rab5 is a marker for early endosomes and a key factor in early endosome transport. The Rab5-mediated endo-lysosomal trafficking pathway is responsible for maturation of early endosomes to late endosomes. Interestingly, we did not observe any difference in the Rab5 staining of *Pfn3^–/–^* testis sections as compared to the *Pfn3^+/–^* and *Pfn3*^+/+^ testis sections (Supplementary Figure 4). This result suggests that in *Pfn3*-deficient mice, the endocytic pathway is not affected.

### RNA-Seq Revealed Alterations in the Expression Levels of Germ Cell Development-Related Genes in Pfn3^–/–^ Mice

**FIGURE 7:**
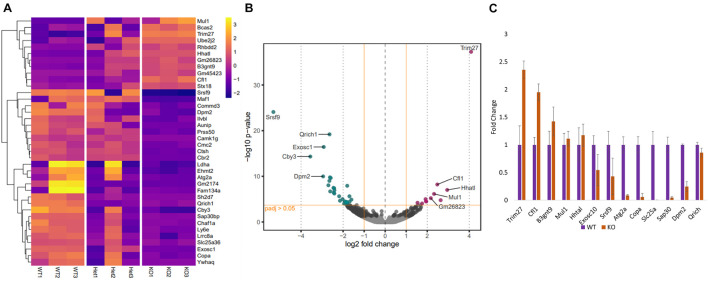
Changes in gene expression profile of *Pfn3*-deficient mice. **(A)** Heat map visualization of top 38 differentially expressed (DE) genes obtained by RNA-seq on *Pfn3^+/+^*, *Pfn3^+/–^*, and *Pfn3^–/–^* testes. **(B)** Volcano plots displaying DE genes for *Pfn3^+/+^* vs. *Pfn3^–/–^* [adjusted *p*-value < 0.05; log2 fold change of expression (LFC) > 1.5]. **(C)** DE genes obtained by RNA-seq were verified by qRT-PCR on *Pfn3^+/+^*, *Pfn3^+/–^*, and *Pfn3^–/–^* testicular RNA (*n* = 3 biological replicates/genotype).

Since the function of PFN3 is only beginning to be understood, we set out to identify whether deletion of *Pfn3* impinges on global gene expression and performed RNA-sequencing on total RNA isolated from testes of *Pfn3^+/+^*, *Pfn3^+/–^*, and *Pfn3^–/–^* mice. RNA-sequencing identified 38 significantly differentially expressed (DE) genes with log2 fold change > 1.5 in *Pfn3^–/–^* as compared to control (Figure 7A). In total, 27 genes were found to be upregulated and 11 genes to be downregulated in *Pfn3^–/–^* testes compared to wild type (Figure 7B). In order to check that the results observed are not skewed due to a defect in spermiogenesis in the PFN3-deficient mice, we tested for expression levels of marker genes indicative for Leydig cells, Sertoli cells, and spermatogonia. Box plots of marker genes for the different cell types are given in the Supplementary Data (Supplementary Figure 5) and reveal that the overall quantity and development of sperm cells are not affected in *Pfn3^–/–^* mice. Tukey’s multiple-comparison ANOVA was used to check for statistics, and all groups showed non-significant difference. Quantitative real-time PCR was used to validate the results for 13 of the DE genes, which are related to male fertility. *Cfl1*, *Trim27*, *B3gnt9*, *Mul1*, and *Hhatl* were upregulated, while *Exosc1*, *Srsf9*, *Atg2a*, *Copa*, *Slc25a36*, *Sap30*, *Dpm2*, and *Qrich1* were downregulated in *Pfn3^–/–^* mice (Figure 7C). These results indicated that deletion of *Pfn3* disrupts the expression of genes involved in actin cytoskeletal dynamics, regulation of autophagy, and mitochondrial and Golgi network structural integrity.

### Autophagic Flux and AMPK/mTOR Signaling Pathway Are Affected in Pfn3-Deficient Mice

**FIGURE 8:**
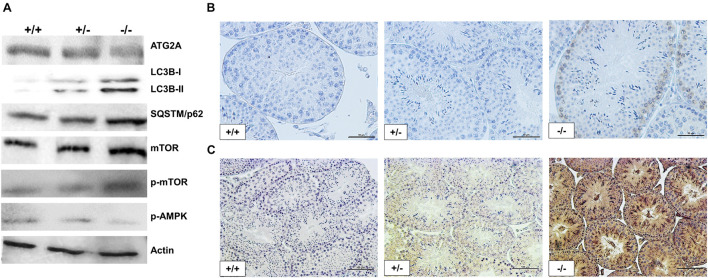
Disruption in autophagic flux and AMPK/mTOR signaling pathway of *Pfn3*-deficient mice. **(A)** Immunoblot analysis against ATG2A, LC3B, SQSTM1, mTOR, phospho-mTOR, and phospho-AMPKα on protein lysates from *Pfn3^+/+^*, *Pfn3^+/–^*, and *Pfn3^–/–^* testes. **(B)** Immunohistochemical staining against phospho-mTOR on *Pfn3^+/+^*, *Pfn3^+/–^*, and *Pfn3^–/–^* testis sections (top row). **(C)** Immunohistochemical staining against LC3B on *Pfn3^+/+^*, *Pfn3^+/–^*, and *Pfn3^–/–^* testis sections (bottom row). Staining of testicular tissue sections from Pfn3^+/+^ (left column), Pfn3^+/–^ (middle column), and Pfn3*^–/–^* (right column) animals is shown. Scale bar = 100 μm.

The RNA-seq analysis revealed *Trim27* as the most upregulated gene in *Pfn3*-deficient mice. The overexpression of *Trim27* leads to activation of AKT signaling (Zhang et al., 2018). AKT in turn is an activator of mTOR (Hahn-Windgassen et al., 2005). Autophagy is regulated by AMPK/mTOR signaling pathways, with AMPK being stimulating and mTOR being repressive (Liang et al., 2018). Next, we were interested to determine whether the signaling pathways downstream of *Trim27* responsible for regulation of autophagy are affected in *Pfn3*-deficient mice. Indeed, immunoblotting showed increased protein levels for mTOR and phospho-mTOR, while the level of phospho-AMPKα was reduced (Figure 8A). Increased level of phospho-mTOR was validated by IHC in *Pfn3*-deficient mice (Figure 8B). So, these data suggest that loss of *Pfn3* leads to an upregulation of *Trim27*, which results in the activation of mTOR signaling (Hahn-Windgassen et al., 2005; Liang et al., 2018; Zhang et al., 2018), causing a decrease in p-AMPKα resulting in the disruption of autophagy. Furthermore, the autophagic gene *Atg2a* was expressed at lower levels in *Pfn3*-deficient mice. *Atg2a* is involved in the phagophore elongation leading to the formation of the autophagosome (Bozic et al., 2020). The elongation step is completed by the conjugation of LC3B, known as microtubule-associated protein and a widely used marker for autophagosomes (Tang et al., 2017). LC3B is a core protein in the autophagic flux where it functions as an autophagic cargo by interacting with an autophagic substrate SQSTM1/p62 (Tang et al., 2017). It is well established that depletion of *Atg2a* results in blocking of autophagic flux leading to accumulation of LC3B and SQSTM1 (Bozic et al., 2020). Therefore, we analyzed LC3B and SQSTM1 protein levels. Interestingly, in *Pfn3*-deficient testes, levels of LC3B were increased as shown by WB and IHC (Figures 8A,C, respectively). We further found an accumulation of SQSTM1 in *Pfn3*-deficient testes using WB (Figure 8A). Quantification of protein levels (Supplementary Figure 6) revealed an increase in LC3B, P62, p-mTOR, and mTOR levels and reduction of ATG2a and AMPK. So, we hypothesized that deletion of *Pfn3* results in upregulation of *Trim27*, which leads to mTOR-mediated inhibition of autophagy hallmarked by lower levels of *Atg2a*. As a consequence, autophagic flux stalls, indicated by accumulation of LC3B and SQSTM1. This might cause the disturbance of the acrosome formation in *Pfn3*-deficient mice.

### PFN3 Interacts With TRIM27

**FIGURE 9:**
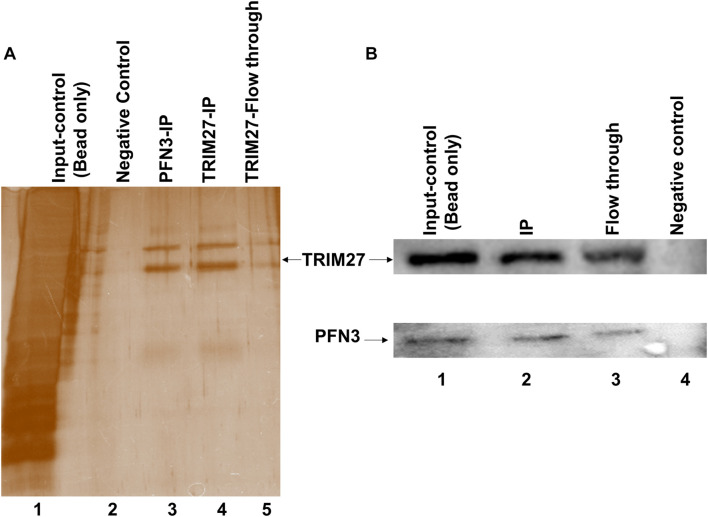
Co-immunoprecipitation using anti-PFN3 and anti-TRIM27 antibody on testis lysates. **(A)** Silver-stained SDS-PAGE; lane 1: input control (protein lysate), lane 2: negative control (only antibody), lane 3: IP using PFN3 antibody, lane 4: IP using PFN3 antibody, and lane 5: flow through. **(B)** Western blot showed PFN3 and TRIM27 proteins. Lane 1: input control. Lane 2: immunoblot of reciprocal IP for TRIM27 (band observed at ∼58 kDa) and PFN3 protein levels (band observed at ∼14 kDa) using anti-PFN3 and anti-TRIM27 antibodies, respectively. Lane 3: flow through. Lane 4: negative control.

Next, we used co-immunoprecipitation (Co-IP) to test whether PFN3 and TRIM27 interact. From whole-testis lysate, a PFN3-specific antibody pulled down TRIM27 (Figure 9A, lane 3) and the IP with a TRIM27 antibody was able to capture and elute PFN3 (not shown). The specificity of the assay was confirmed by using protein extraction buffer as a negative control (Figure 9A, lane 2); whole-testis lysate was used as an input control (Figure 9A, lane 1). To validate this interaction, reciprocal antibodies on immunoblot (Figure 9B) showed PFN3 (∼14 kDa) and TRIM27 (∼58 kDa) proteins from input control (lane1), Co-IP (lane 2), flow through (lane 3), and negative control.

### Pfn3-Deficient Mice Exhibit Increased Protein Levels of ADF/CFL Variants Compared to WT

**FIGURE 10:**
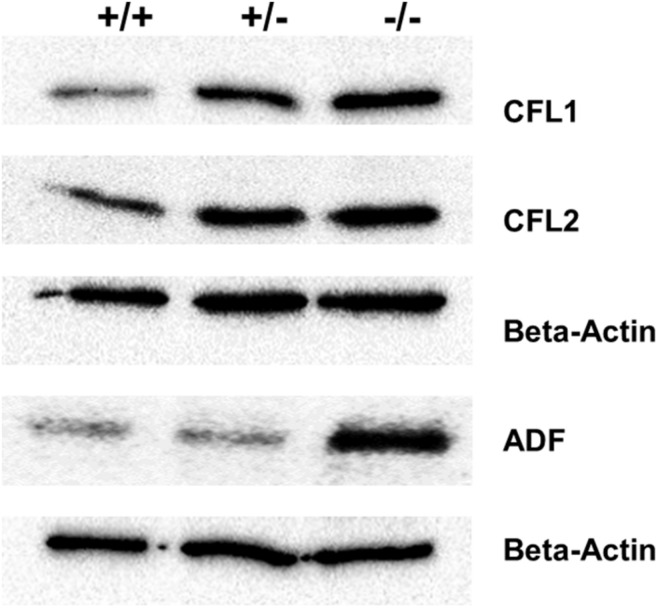
Immunoblotting against ADF, CFL1, and CFL2 on protein lysates from *Pfn3^+/+^*, *Pfn3^+/–^*, and *Pfn3^–/–^* testes.

Furthermore, the expression of cofilin1 (*Cfl1*) was upregulated in *Pfn3*-deficient testes. We decided to check protein levels using WB for the cofilin traditional proteins (CFL1, CFL2, and ADF) known as actin-binding proteins. The level of ADF protein was increased in *Pfn3^–/–^* testes, while CFL1 and CFL2 (Figure 10) protein levels were already increased in heterozygous testes.

### PFN3 Binding Protein Is Lost in Nuclear Fraction of Sperm

PFN3 is detected in a complex with ARPM1, specifically in the sperm nucleus, however, not in the cytoplasm (Hara et al., 2008). In sperm cytoplasm of *Pfn3^–/–^* mice, a moderate signal of ARPM1 can still be detected (Supplementary Figure 7A). We performed WB using anti-ARPM1antibody on proteins isolated from cytoplasmic and nuclear fractions of both testes and sperm. Western blot showed that ARPM1 could not be detected in the nuclear fraction of *Pfn3*-deficient testes and sperm, while cytoplasmic ARPM1 protein levels in testes are slightly reduced in *Pfn3*-deficient mice (Supplementary Figure 7A).

These findings were further confirmed by immunohistochemistry; the nucleus of spermatozoa in testes of *Pfn3*-deficient mice were devoid of ARPM1 (Supplementary Figure 7B). This finding suggests that loss of PFN3 destabilizes the PFN3–ARPM1 complex, leading to loss of ARPM1 in the nuclei of *Pfn3*-deficient sperm.

### F-Actin Organization Is Not Altered in Pfn3-Deficient Sperm

*Pfn3* binds to actin monomers and plays a role in actin polymerization. Actin is mainly located in the mid-piece of sperm flagellum, suggesting a role in sperm motility, elasticity, and membrane integrity. F-actin is present as a helical structure (Gervasi et al., 2018), which is in parallel with the organization of the mitochondrial sheath in mouse sperm (Amaral et al., 2013). We wanted to see whether the *Pfn3* deletion affects the actin cytoskeleton organization in the sperms. Phalloidin ATTO647 fluorescence staining revealed that actin assembly was not altered in the mid-piece of *Pfn3^–/–^* sperm (Supplementary Figure 8).

### Abnormal Manchette Development in Pfn3-Deficient Mice

We found that PFN3 localized in the manchette complex of the developing spermatid. The manchette is a transient structure on the posterior part of the sperm head and is involved in nuclear shaping and protein transport for sperm flagellum formation. The manchette is first detected in step 8, when round spermatids start to elongate by nuclear polarization and the nuclear shape changes from spherical to slightly elongated. After shaping the sperm head, the manchette disappears at step 13 (Okuda et al., 2017).

**FIGURE 11:**
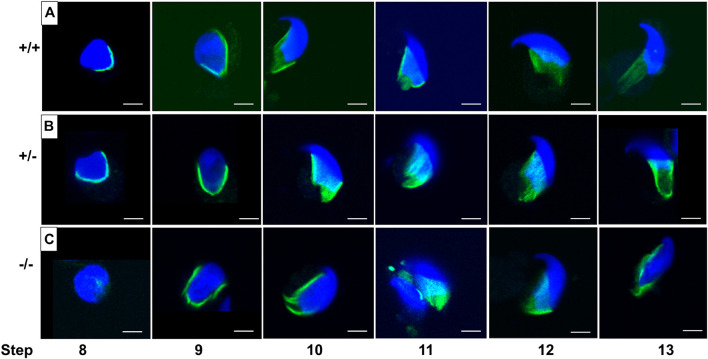
Manchette structure stained by using α-tubulin antibody (green) on germ cell population isolated from **(A)** *Pfn3^+/+^*, **(B)** *Pfn3^+/–^*, and **(C)** *Pfn3^–/–^* testes (*n* = 3/genotype). Nuclei were stained with DAPI (blue). Scale bar = 20 μm.

In order to investigate whether the abnormal head morphology of *Pfn3*-deficient sperm was due to the alteration in manchette structure or formation, we performed an immunofluorescence staining using alpha tubulin for step-by-step comparison on the germ cell population isolated from testes. Tubulin staining revealed that in WT and heterozygous spermatids (Figures 11A,B), manchette was forming the proper skirt-like structure while the manchette was not properly covering and constricted overly at the posterior region in *Pfn3*-deficient round spermatids (Figure 11C). This abnormal manchette development is more obvious in the later steps of development (steps 9–13) in *Pfn3*-deficient spermatids. We conclude that deletion of PFN3 leads to disruption of manchette formation, which in turn contributes to the abnormal shape of the sperm head in *Pfn3*-deficient sperm.

### Pfn3-Deficient Sperm Display Flagellar Deformities

**FIGURE 12:**
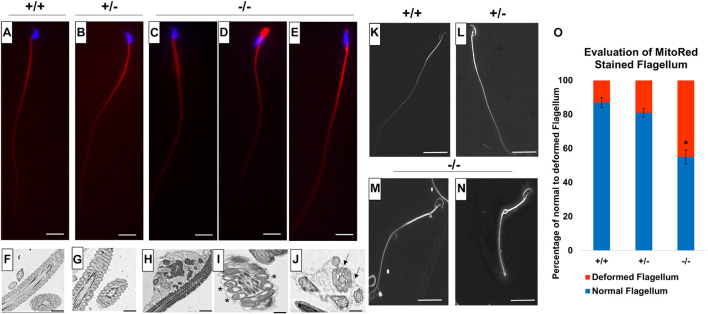
Flagellum analysis on mature sperm cells from *Pfn3^+/+^*, *Pfn3^+/–^*, and *Pfn3^–/–^* mice. Mito Red staining (red) of sperm flagellum on **(A)** *Pfn3^+/+^*; **(B)** *Pfn3^+/–^*; and **(C–E)** *Pfn3^–/–^* sperm cells isolated from cauda epididymis. Scale bar = 10 μm. Ultrastructural analysis using TEM on **(F)** *Pfn3^+/+^*; **(G)** *Pfn3^+/–^*; and **(H–J)** *Pfn3^–/–^* sperm cells isolated from cauda epididymis. Vacuolated mitochondria are shown by asterisk, and fibrous sheet that contained more than one axoneme–mitochondrial complex is shown by arrows. Scale bar = 5 μm. Surface analysis using SEM on **(K)** *Pfn3^+/+^*, **(L)** *Pfn3^+/–^*, and **(M**,**N)** *Pfn3^–/–^* mature sperm cells isolated from cauda epididymis. Scale bar = 50 μm. **(O)** Statistical analysis of Mito Red-stained flagellum of *Pfn3^+/+^*, *Pfn3^+/–^*, and *Pfn3^–/–^* sperm cells. Data is presented as mean ± SD using ANOVA (Tukey’s *post hoc*) (*p < 0.05).

Since we detected low sperm motility in *Pfn3*-deficient mice, we analyzed sperm flagella structure. We used Mito Red immunostaining to assess the mitochondrial sheath in the mid-piece of sperm flagella. While Mito Red uniformly stained the mitochondrial sheath in spermatozoa of WT (Figure 12A) and heterozygous (Figure 12B), *Pfn3*-deficient sperm flagella showed a variety of aberrations spanning from abnormally thick mid-piece (Figure 12C), slightly tapered bent thick neck (Figure 12D), and cytoplasmic droplets with amorphous sperm heads (Figure 12E). This suggests that loss of PFN3 resulted in flagellar deformities due to mitochondrial disorganization.

Next, we performed TEM on mature sperm isolated from cauda epididymis to analyze whether the mid-piece of sperm have normal axonemal and mitochondrial structure. TEM of *Pfn3*-deficient sperm showed several ultrastructural defects, like plasma membrane not covering uniformly the mitochondrial sheet (Figure 12H), disorganized or vacuolated mitochondria (Figure 12I, asterisk), axonemal fibrous sheet enclosed two or more mitochondria and axonemal flagellar complex (Figure 12J) compared to WT (Figure 12F), and heterozygous sperm ultrastructure (Figure 12G). Scanning electron microscopy (SEM) showed similar sperm abnormalities in *Pfn3*-deficient mice, thick sperm mid-pieces with distal cytoplasmic droplets (Figures 12M,N) compared to WT (Figure 12K) and heterozygous sperm (Figure 12L). This result correlates with the previous (Mito Red and TEM ultrastructural sperm analysis) findings. These findings showed that sperm from *Pfn3*-deficient mice display an abnormal morphology of mitochondrial and axonemal fibrous sheet. Statistical analysis revealed that the percentage of deformed sperm flagella in *Pfn3*-deficient mice (∼55%) compared to WT and heterozygous sperms was significantly higher (Figure 12O). We speculate that these defects contribute to the reduced sperm motility. Altogether, our results indicate that loss of PFN3 located in mitochondria resulted in sperm flagellar defects and further support a cytoplasm removal defect.

## Discussion

*Pfn3* has multiple physiological roles in sperm formation and function. In this study, we have shown that deletion of *Pfn3* results in male subfertility hallmarked by reduced sperm count/vitality with sperm displaying type II globozoospermia. *Pfn3*-deficient mice display an impaired acrosome biogenesis followed by malformed acrosomal covering on mature spermatozoa including cytoplasm removal defects, abnormal manchette development contributing to amorphous head shape of sperm, and flagellar deformities resulting in reduced sperm motility. In addition, loss of *Pfn3* disturbs the morphology of Golgi sub-domains, resulting in abnormal formation of Golgi-derived vesicles. Furthermore, we found by co-IP that PFN3 interaction with TRIM27 plays a role in autophagy for acrosome development. Mechanistically, we found a deregulation of autophagy master regulators (*Trim27*, AMPK, mTOR, *Atg2a*, LC3B, and SQSTM1), which seem to relate the disruption of acrosome formation in *Pfn3*-deficient germ cells.

The first and foremost effect of deleting *Pfn3* was impaired acrosome development due to defective vesicle transport from Golgi. Golgi complexes were disorganized and not oriented correctly, leading to an impaired post Golgi trafficking. Acrosome labeling in *Pfn3*-deficient testes revealed that acrosome biogenesis was affected in the Golgi phase of development. The underlying defect was the failure of proacrosomal granule formation and fusion. Loss of *Pfn3* affected the transport of vesicles released from Golgi complexes in *Pfn3*-deficient mice. It is known that profilin1 is found in the Golgi compartment (Dong et al., 2000) and profilin2 is associated with proteins that play a role in membrane trafficking (Gareus et al., 2006).

The formation of the acrosome uses elements of the autophagy machinery that are involved in the fusion/transportation of Golgi-derived proacrosomal vesicles (Wang et al., 2014). We demonstrate that deletion of *Pfn3* leads to upregulation of *Trim27*, which leads to the activation of mTOR. This, in turn, leads to repression of AMPK. Together, higher mTOR and lower AMPK lead to an attenuation of autophagy hallmarked by lower levels of *Atg2a.* As a consequence, autophagic flux stalls, indicated by accumulation of LC3B and SQSTM1. This causes the developmental arrest of the acrosome formation in *Pfn3*-deficient mice. Disruption of autophagic flux leading to failure of proacrosomal granule formation is also reported in mice deficient for *Sirt1* (Liu et al., 2017) and *Atg7* (Wang et al., 2014). Interestingly, in *Atg7* mutants, LC3B levels are increased, but AMPK and mTOR levels remain unaffected (Wang et al., 2014). However, in *Sirt1* mutants, autophagic flux is partially disrupted by the accumulation of acetylated LC3B in the nucleus (Liu et al., 2017). This suggest that ATG7 and SIRT1 act further downstream in the autophagic cascade compared to *Pfn3*.

We demonstrate that loss of *Pfn3* leads to an upregulation of *Trim27*. So, we speculate that *Pfn3* directly or indirectly interacts with or is tethered to *Trim27* to modulate its activity during spermiogenesis. In fact, the interaction was demonstrated using co-immunoprecipitation. So, we hypothesize that a lack of *Pfn3* unleashes *Trim27* leading to i) enhanced expression of *Trim27*, which initiates a cascade resulting in ii) impaired acrosome development.

In addition, acrosome labelling and evaluation of transmission electron microscopy showed malformed/fragmented acrosomes in 50–60% of mature epididymal *Pfn3*-deficient sperm. Besides defective acrosome morphology of mature spermatozoa, 70% of *Pfn3*-deficient sperms showed amorphous head shape lacking the typical hook area and circularity. We demonstrated that the development of the manchette is disturbed in *Pfn3*-deficient sperm. Defective manchette development leading to abnormal shaping of sperm head is also reported in *Katnb1*, *Sun4*, *Lrguk1*, *Kif3A*, *Hook1*, and *Kash* mutants. Intriguingly, these genes interact with the microtubule network and the proteins are localized in the microtubular manchette (Mendoza-Lujambio et al., 2002; O’Donnell et al., 2012; Lehti et al., 2013; Gunes et al., 2020). The manchette is connected to the nucleus by fuzzy material/linkers, which indicates that manchette and nucleus possess a structural relationship through which they exert forces on each other for the shaping of sperm head (Russell et al., 1991). This suggests that loss of microtubular proteins in the manchette disturbs the structural relationship between manchette and nucleus resulting in abnormal sperm head development. The fact that PFN3 is localized to the microtubules of the manchette, and loss of PFN3 results in manchette deformities, suggests that PFN3 contributes to the organization and remodeling of the manchette for sperm head shaping.

The fact that the sperm count is significantly reduced is most likely due to the observed motility defect.

Further, *Pfn3*-deficient sperm showed significant reduction in progressive motility as well as cytoplasmic removal defects. Vacuolated mitochondria result in the deformities in the flagellum leading to the reduced motility. Sperm motility is the outcome of flagellar movement of sperm tail, gained by ATP-driven energy produced by mitochondrion located in sperm mid-piece (Tourmente et al., 2015). Sperm flagellum deformities lead to poor sperm motility and abnormal flagellum structure, as seen in other mouse KO models such as TSSK4, QRICH2, and CABYR (Lehti and Sironen, 2017; Shen et al., 2019). This suggests that deformities of the flagellum led to the reduced motility in *Pfn3^–/–^* mice, however, caused by a yet unexplained mechanism.

To our surprise, we observed unaltered F-actin organization in *Pfn3*-deficient sperm. This suggests that the role of *Pfn3* in actin polymerization is rather minor.

Hara et al. (2008) showed that, in the nuclei of spermatids, ARPM1 binds to PFN3, while cytoplasmic ARPM1 does not. The ARPM1–PFN3 complex contributes to spermatid head shaping (Hara et al., 2008). We found that a lack of PFN3 led to the loss of ARPM1 in spermatid nuclei. We hypothesized that PFN3 is required for the stabilization or localization of ARPM1 in nuclei of the spermatids. So, the abnormal sperm nuclear morphology observed in *Pfn3*-deficient mice might be a consequence of a lack of testes-specific PFN3–ARPM1 complex. In addition, ARPM1 protein was detected in the cytoplasm of testes since ARPM1–PFN3 complex is only restricted to sperm nucleus, which further confirms the findings of Hara et al. (2008).

Further, *Srsf9*, *Slc25a36*, *Prss50*, and *Copa* are downregulated in our *Pfn3*-deficient mice. Bansal et al. (2015) reported that patients with reduced sperm motility known as asthenozoospermia display lower levels of *Srsf9*. Additionally, *Slc25a36* is a member of the solute carrier super family and is known to regulate mitochondrial function. It is reported by Xin et al. (2019) that *Slc25a36* deficiency led to impaired mitochondria and decreased mitochondrial membrane potential. PRSS50 and COPA are involved in male fertility and Golgi trafficking, respectively, (Sleutels et al., 2012; Custer et al., 2019). Downregulation of PRSS50 resulted in reduced male fertility (Sleutels et al., 2012). Moreover, knockdown of COPA impairs Golgi–ER trafficking (Tang et al., 2015). We reported severely reduced male fertility and sperm motility, malformed mitochondria, and disturbed Golgi-derived vesicles in *Pfn3*-deficient mice. In addition, we observed that *Mul1* is upregulated in *Pfn3*-deficient testes. *Mul1* is essential for maintaining the mitochondrial morphology (Li J. et al., 2015). We found vacuolated mitochondria in sperm flagellum of *Pfn3*-deficient mice. These findings suggest that loss of *Pfn3* results in differentially expressed genes leading to reduced sperm motility and flagellar deformities.

**FIGURE 13:**
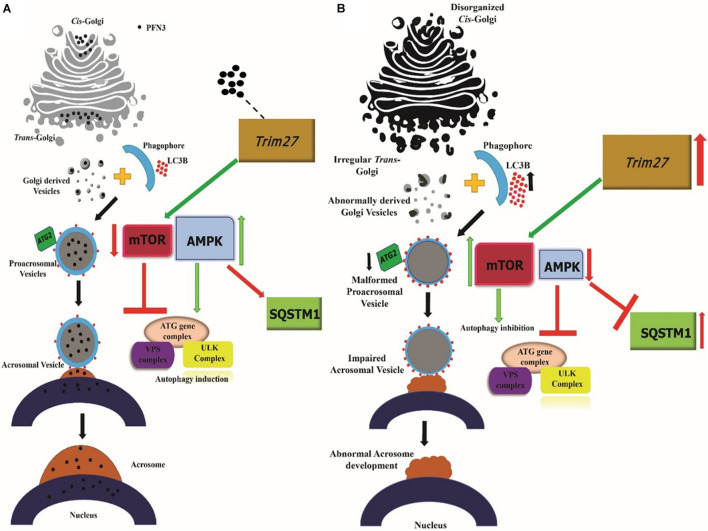
Working hypothesis on the PFN3 role in acrosome biogenesis. **(A)** Schematic illustration of the *Pfn3* presence in Golgi network responsible for proacrosomal formation associated with the autophagy mechanism. **(B)** Schematic illustration depicting the disrupted autophagy mechanism and acrosome formation in the absence of *Pfn3*. Black dots = PFN3.

Taken together, our findings demonstrate that *Pfn3* affects multiple processes during spermiogenesis. We summarize our findings related to the role of PFN3 in vesicle transport from Golgi to nucleus for acrosome biogenesis in Figure 13. A lack of PFN3 causes a disruption of Golgi sub-domains leading to impaired acrosome biogenesis. On a molecular level, loss of PFN3 leads to upregulation of *Trim27*, resulting in deregulation of mTOR and AMPK signaling, leading to a disruption of autophagic flux. Furthermore, we show that loss of *Pfn3* causes abnormal manchette development and loss of ARPM1 in sperm nucleus. We detected vacuolated mitochondria in the flagellum. This might lead to reduced sperm motility in *Pfn3*-deficient mice. In conclusion, our study highlights the requirement of *Pfn3* during spermiogenesis specifically in acrosome biogenesis and adds this gene to the growing catalog of genes potentially involved in human male infertility.

## Data Availability Statement

RNA-seq data from this study have been submitted to the NCBI Gene Expression Omnibus (GEO; https://www.ncbi.nlm.nih.gov/geo/) under accession no. GSE171068.

## Ethics Statement

The animal study was reviewed and approved by Landesamt für Natur, Umwelt und Verbraucherschutz, North Rhine-Westphalia, approval ID: AZ84- 02.04.2013.A429.

## Author Contributions

NU and HS conceived and designed the project. NU generated the knockouts and performed the detailed experimental analyses. SP and DoS performed basic analyses. LA contributed to RNA-seq analysis. DeS contributed to IF staining of PFN3. KL and GK contributed to scanning electron microscopy. DoS carried out STED microscopy. NU wrote the manuscript. HS supervised the manuscript. All authors contributed to the article and approved the submitted version.

## Conflict of Interest

The authors declare that the research was conducted in the absence of any commercial or financial relationships that could be construed as a potential conflict of interest.

## Publisher’s Note

All claims expressed in this article are solely those of the authors and do not necessarily represent those of their affiliated organizations, or those of the publisher, the editors and the reviewers. Any product that may be evaluated in this article, or claim that may be made by its manufacturer, is not guaranteed or endorsed by the publisher.

## Footnotes

1
http://crispr.mit.edu

2
https://ftp.ensembl.org/
