# Supplementary material for: Loss of Profilin3 Impairs Spermiogenesis by Affecting Acrosome Biogenesis, Autophagy, Manchette Development and Mitochondrial Organization
Source: Front Cell Dev Biol. 2021 Nov 4;9:749559. doi: 10.3389/fcell.2021.749559 (PMC8632698; doi:10.3389/fcell.2021.749559)
Supplement: Supplementary file 1 [file Data_Sheet_1.PDF]

Loss of profilin3 impairs Spermiogenesis by affecting acrosome biogenesis, autophagy, manchette development and mitochondrial organization

Naila Umer[1], Lena Arevalo[1], Sharang Phadke[1,3], Keerthika Lohanadan[4], Gregor Kirfel[4], Dominik Sons[5], Sophia Denise[6], Walter Witke[6], Hubert Schorle[1*].

Supplementary data

Supplementary Figure. 1

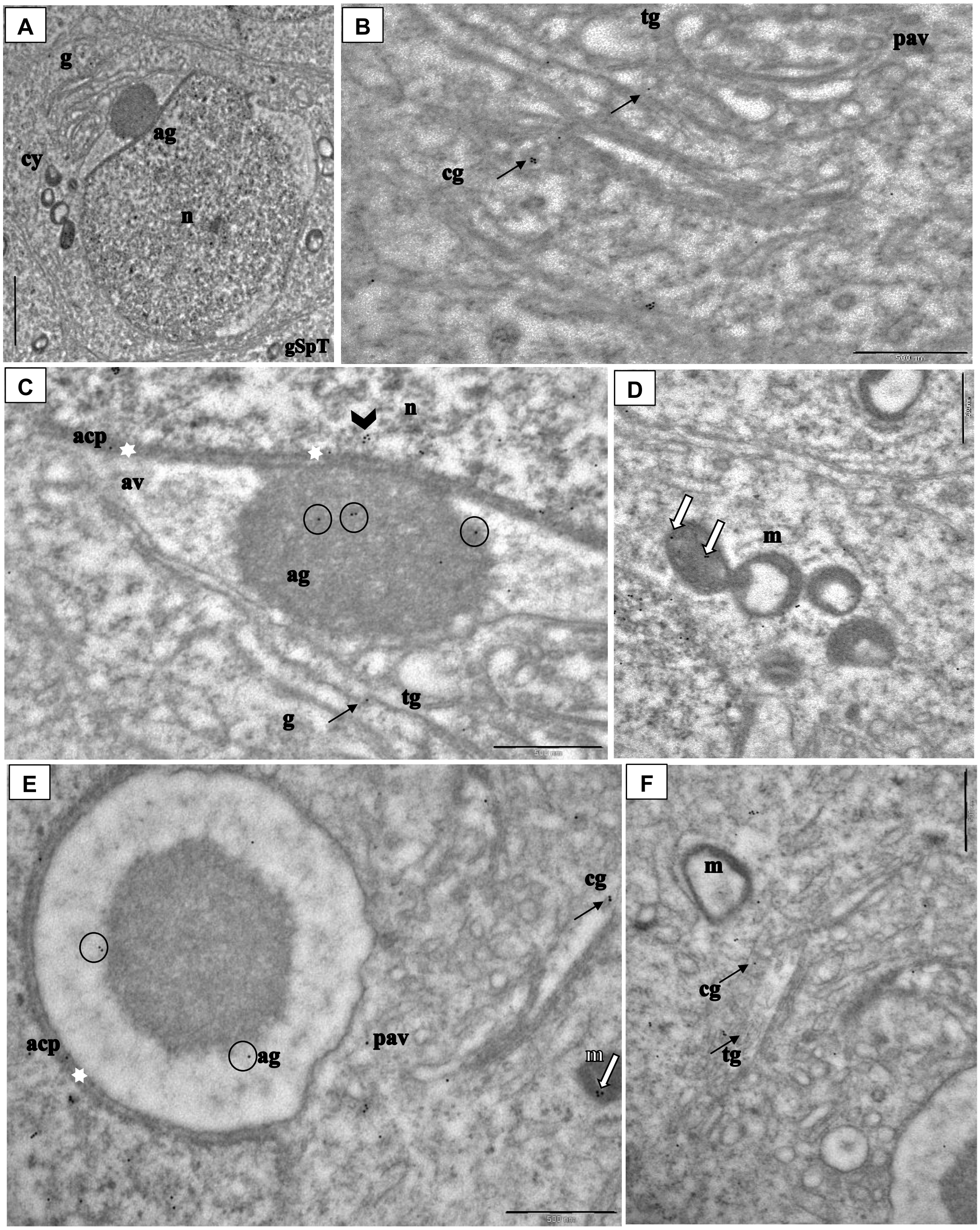

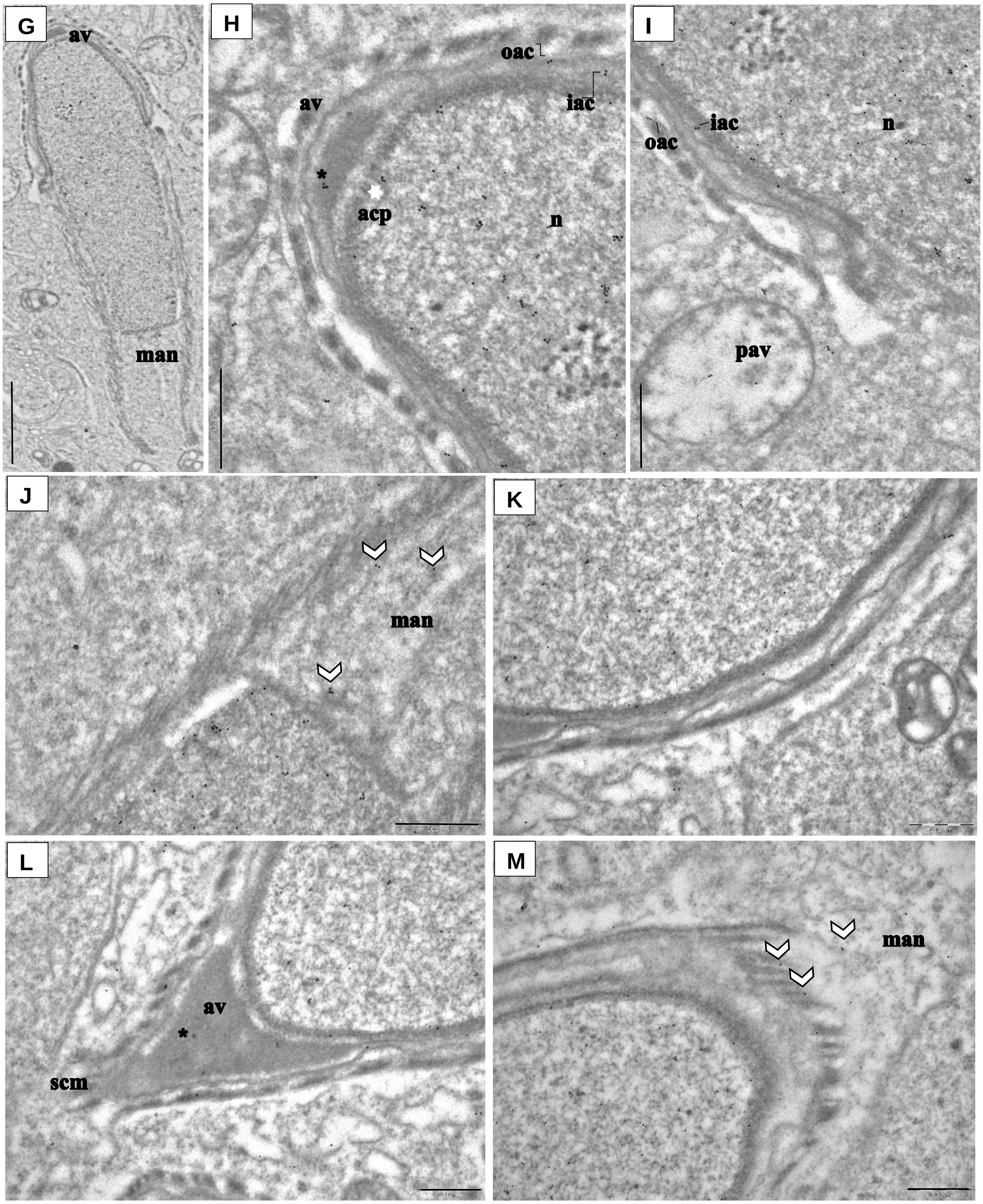
G
av
man
H
oac
iac
av
*
acp
n
I
iac
oac
n
pav
J
man
K
L
av
*
scm
M
man

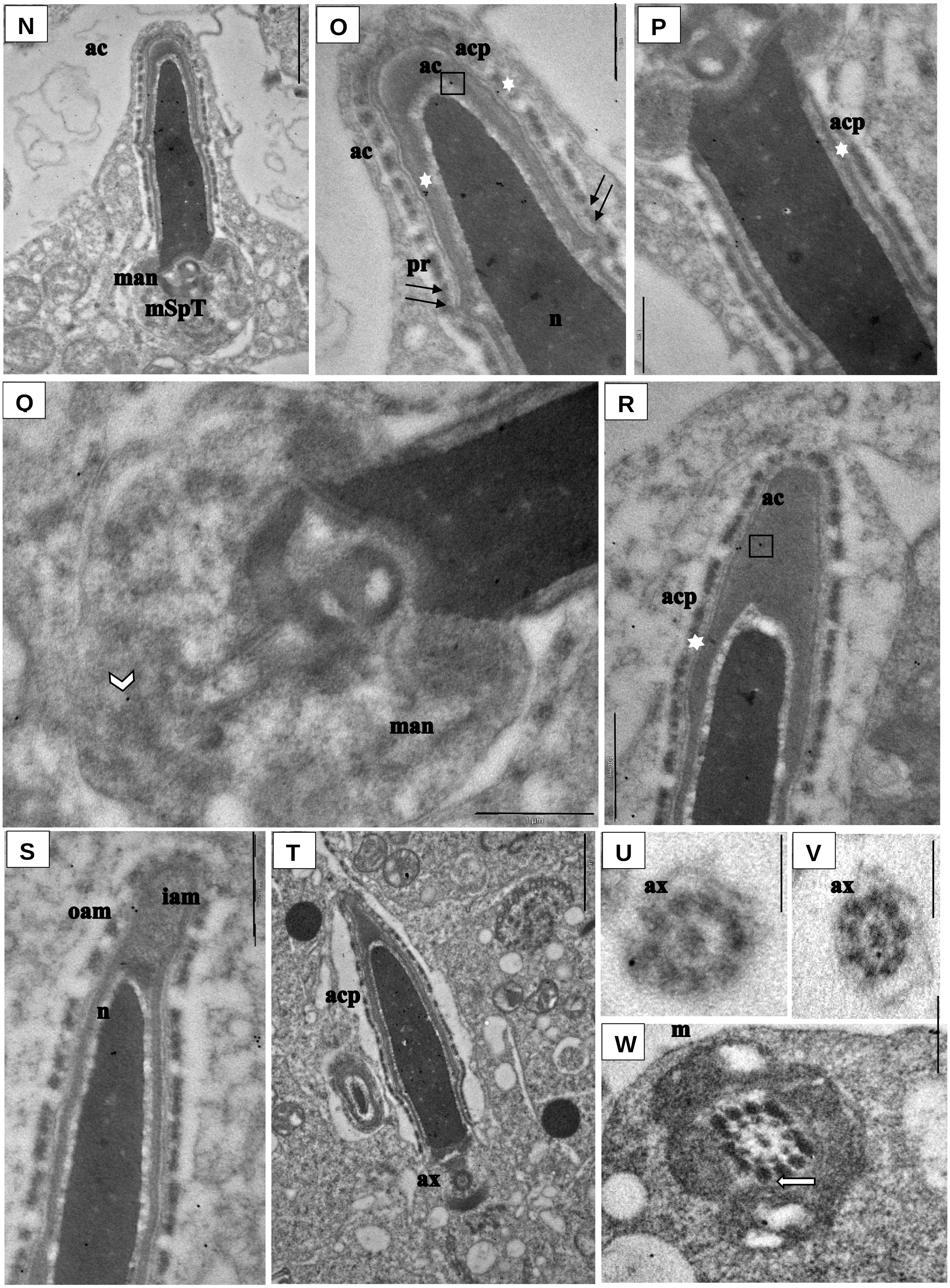
N
ac
man
mSpT
O
acp
ac
ac
pr
n
P
acp
Q
man
1 µm
R
ac
acp
S
oam
iam
n
T
acp
ax
U
ax
V
ax
W
m

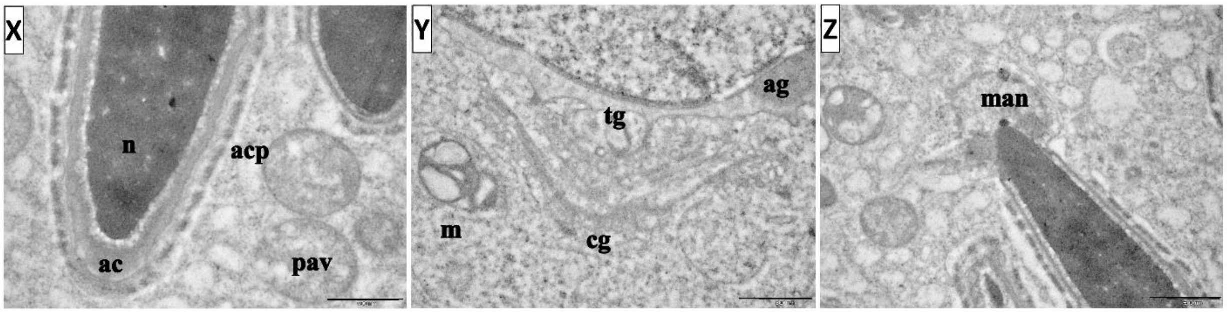

Supplementary Figure 1 (A-F) In Golgi phase, **(G-M)** In Cap phase, (**N-W)** In Maturation phase, PFN3 is localized in various cellular compartments. (**X-Z)** Negative control. **g**=Golgi, **cy**=cytoplasm, **ag**=acrosomal granule, **n**=nucleus, **gSpT**=spermatid in Golgi phase, **cg**=cis-Golgi, **tg**=trans-Golgi, **pav**=proacrosomal vesicle, **acp**=acroplaxome, **m**=mitochondria, **av**=acrosomal vesicle, **man**=manchette, **oac**=outer acrosomal membrane, **iac**=inner acrosomal membrane, **scm**=sertoli cell membrane, **ac**=acrosome, **pr**=perinuclear ring, **mSpT**=spermatid mid piece, **ax**= axoneme. Details of indications described in the result part. Scale bar = 1 μm & 500 nm.

Supplementary Figure. 2

S2A

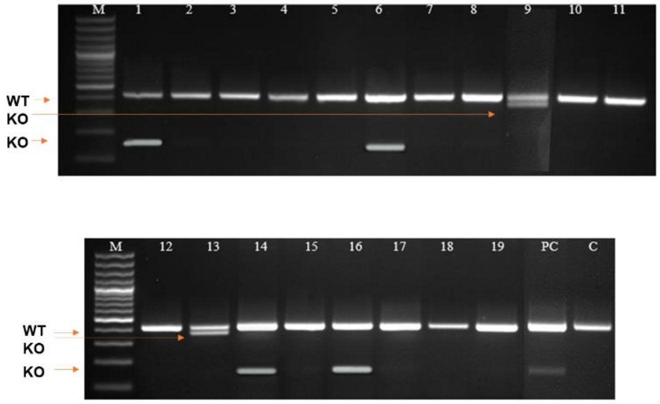

S2B

```
WT            ATGAGTGACTGGAAGGGCTACATCAGTGCAGTGCTGCGGGATCAGCGGATCGATGACGTG   60
Pfn3-254bp    ATGAGTGACTGGAAGGGCTACATCAGTGCAGTGCTGCGGGATCAGCGGTGTGCTCAATAA   60
              ************************************************   * * *

WT            GCTATCGTGGGCCACTCGGACAATCGCTGTGTGTGGGCATCACGGCCTGGGGTCTGCTG   120
Pfn3-254bp    G-----------------------------------------------------------   61
              *

WT            GCTGCCATCTCCCCGCAGGAGGTGGGTGTGCTCACCGGGCCAGACCGGCACACCTTTCTG   180
Pfn3-254bp    ------------------------------------------------------------   61

WT            CAGACCGGTCTGAGCGTGGCAGGCCGCCGCTGCTGCGTTATCCGTGACTACCTGCTGGCC   240
Pfn3-254bp    ------------------------------------------------------------   61

WT            GAGGGTGACGGCGTACTGGATGCGCGCACCAAGGGGCTAGACGGGCGTGCAATCTGTGTG   300
Pfn3-254bp    ------------------------------------------------------------   61

WT            GGCCACACGCCACGGGCGCTCCTGGTGCTCATGGGCAGACGAGGCGTGCATGGAGGCATC   360
Pfn3-254bp    ---------------ACAGTGCACGATCTGATTGGTGGGCTGCGTGAGCAGTGCTCTTAG   106
                              *  * *  *  ** ** **  * *   * * ***  *

WT            CTCAATAAGACAGTGCACGATCTGATTGGTGGGCTGCGTGAGCAGTGCTCTTAG  414
Pfn3-254bp    CTCAATAAGACAGTGCACGATCTGATTGGTGGGCTGCGTGAGCAGTGCTCTTAG  160
              ******************************************************
```

```
Pfn3          ATGAGTGACTGGAAGGGCTACATCAGTGCAGTGCTGCGGGATCAGCGGATCGATGACGTG   60
Pfn3-41bp     ATGAGTGACTGGAAGGGCTACATCAGTGCAGTGCTGCGGACTCAGTGCAGTGCTGCGGGA   60
              ***************************************  **** * *  * **  *

Pfn3          GCTATCGTGGGCCACTCGGACAATCGCTGTGTGTGGGCATCACGGCCTGGGGTCTGCTG   120
Pfn3-41bp     TCAGCGGATCG--------------------------------------ATGA---CGTG   79
               *    *   *                                        *      **

Pfn3          GCTGCCATCTCCCCGCAGGAGGTGGGTGTGCTCACCGGGCCAGACCGGCACACCTTTCTG   180
Pfn3-41bp     ATGAGTGACTGGAAGGGCTACATCAGTGCAGTGCTGCGGGATCAGCGGATCGATGACGTG   139
                      **    *    *  *  ***   *     **    * ***  *       **

Pfn3          CAGACCGGTCTGAGCGTGGCAGGCCGCCGCTGCTGCGTTATCCGTGACTACCTGCTGGCC   240
Pfn3-41bp     CAGACGGGTCTGAGCGTGGCAGGCCGCCGCTGCTGCGTTATCCGTGACTACCTGCTGGCC   199
              ***** ******************************************************

Pfn3          GAGGGTGACGGCGTACTGGATGCGCGCACCAAGGGGCTAGACGGGCGTGCAATCTGTGTG   300
Pfn3-41bp     GAGGGTGACGGCGTACTGGATGCGCGCACCAAGGGGCTAGACGGGCGTGCAATCTGTGTG   259
              ************************************************************

Pfn3          GGCCACACGCCACGGGCGCTCCTGGTGCTCATGGGCAGACGAGGCGTGCATGGAGGCATC   360
Pfn3-41bp     GAGGGTGACGGCGTACTGGATGCGCGCACCAAGGGGCTAGACGGGCGTGCAATCTGTGTG   319
              *                *     *     ** ***   *   **           *  *

Pfn3          CTCAATAAGACAGTGCACGATCTGATTGGTGGGCTGCGTGAGCAGTGCTCTTAG   414
Pfn3-41bp     CTCAATAAGACAGTGCACGATCTGATTGGTGGGCTGCGTGAGCAGTGCTCTTAG   373
              ******************************************************
```

```
WT            ATGAGTGACTGGAAGGGCTACATCAGTGCAGTGCTGCGGGATCAGCGGATCGATGACGTG   60
Pfn3-29bp     -TGAGTGACTGGAAG-------------------GGCTACATCAGTGCAGTGCTGCCTTG   40
               **************                    **   ***** * *  * ** * **

WT            GCTATCGTGGGCCACTCGGACAATCGCTGTGTGTGGGCATCACGGCCTGGGGTCTGCTG   120
Pfn3-29bp     GCTATCGGGGGCCACTCGGACAATCGCTGTGTGTGGGCGTCAGGGCCTGGGGTCTGCTG   100
              ******* ****************************** *** *****************

WT            GCTGCCATCTCCCCGCAGGAGGTGGGTGTGCTCACCGGGCCAGACCGGCACACCTTTCTG   180
Pfn3-29bp     GCTGGCATCTCCCCGCCTGACGTGGGTGTGCTCACCGGGCCAGACCGGCACACCTTTCTG   160
              **** ***********  ** ***************************************

WT            CAGACCGGTCTGAGCGTGGCAGGCCGCCGCTGCTGCGTTATCCGTGACTACCTGCTGGCC   240
Pfn3-29bp     CCTACCGGCCTGAGCGTGGCAGGCCGCCGCTGCTGCGTTGTCCGTGACTACCTGCTGGCC   220
              *  ***** ****************************** ********************

WT            GAGGGTGACGGCGTACTGGATGCGCGCACCAAGGGGCTAGACGGGCGTGCAATCTGTGTG   300
Pfn3-29bp     CCTACCGGCCTGAGCGTGGCAGGCCGCCGCTGCT--------GCGTTGTCCGTGACTACC   272
                    * *       ***  *  ***  *            * *    *  *   *

WT            GGCCACACGCCACGGGCGCTCCTGGTGCTCATGGGCAGACGAGGCGTGCATGGAGGCATC   360
Pfn3-29bp     TGCTGGCCGCCACGGGCGCTCCTGGTGCTCATGGGCAGACGAGGCGTGCCTGGAGGCATC   332
               **    ****************************************** **********

WT            CTCAATAAGACAGTGCACGATCTGATTGGTGGGCTGCGTGAGCAGTGCTCTTAG   414
Pfn3-29bp     CTCAATAACAAAGTGCACGATCTGATTGCTGGGCTGCGAGAGCGCTGCTCTTA-   385
              ******** * ***************** ********* ****  ********
```

S2C

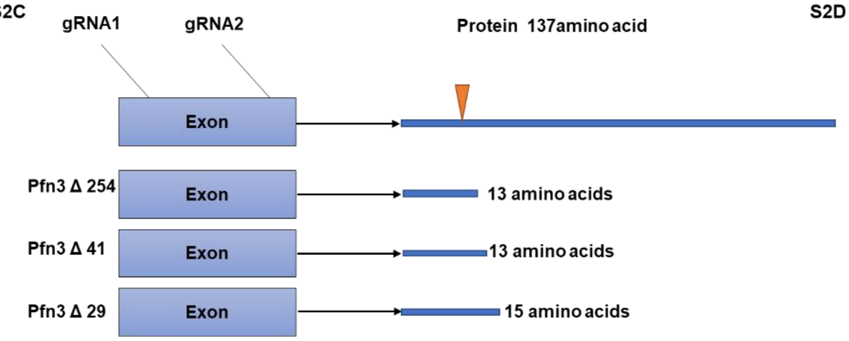
gRNA1
gRNA2
Protein 137amino acid
Exon
Pfn3 Δ 254
Exon
13 amino acids
Pfn3 Δ 41
Exon
13 amino acids
Pfn3 Δ 29
Exon
15 amino acids

S2D

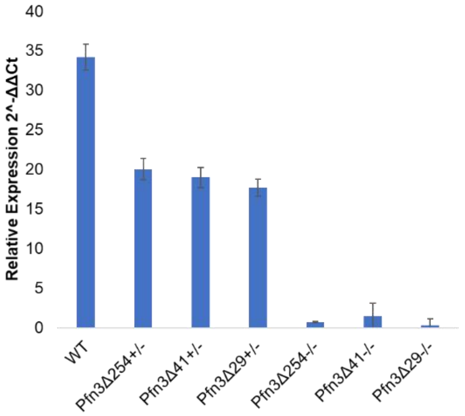
Relative Expression 2^-ΔΔCt
40
35
30
25
20
15
10
5
0
WT
Pfn3Δ254+/-
Pfn3Δ41+/-
Pfn3Δ29+/-
Pfn3Δ254-/-
Pfn3Δ41-/-
Pfn3Δ29-/-

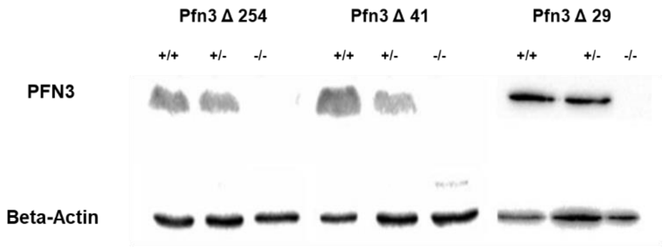
Pfn3 Δ 254
Pfn3 Δ 41
Pfn3 Δ 29
+/+
+/-
-/-
PFN3
Beta-Actin

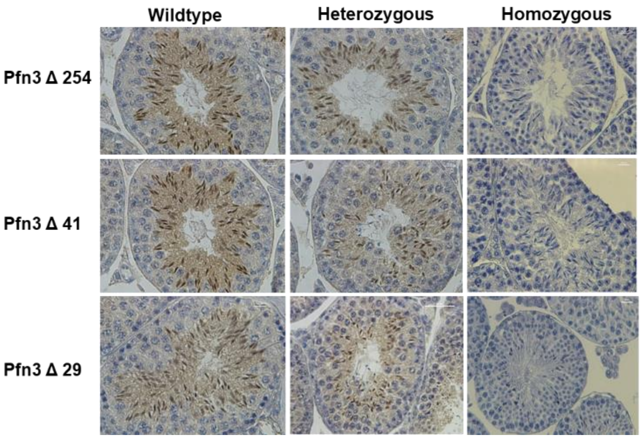
Wildtype
Heterozygous
Homozygous
Pfn3 Δ 254
Pfn3 Δ 41
Pfn3 Δ 29

S2E

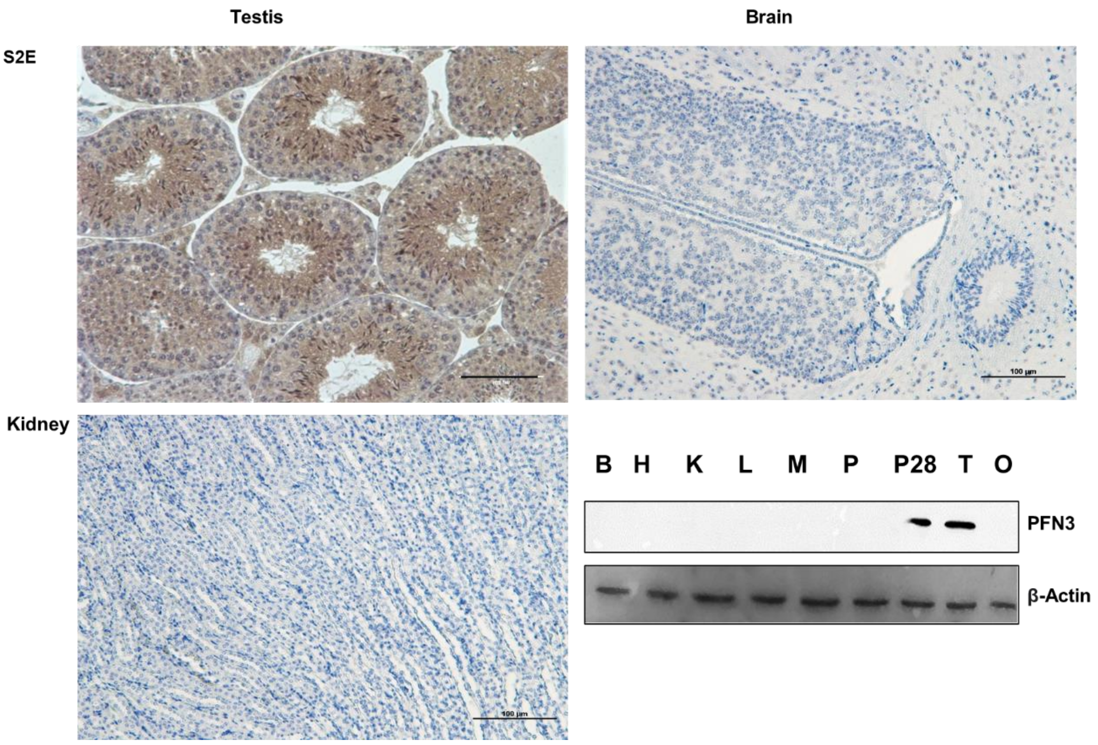
Testis
Brain
Kidney
B
H
K
L
M
P
P28
T
O
PFN3
β-Actin

S2F

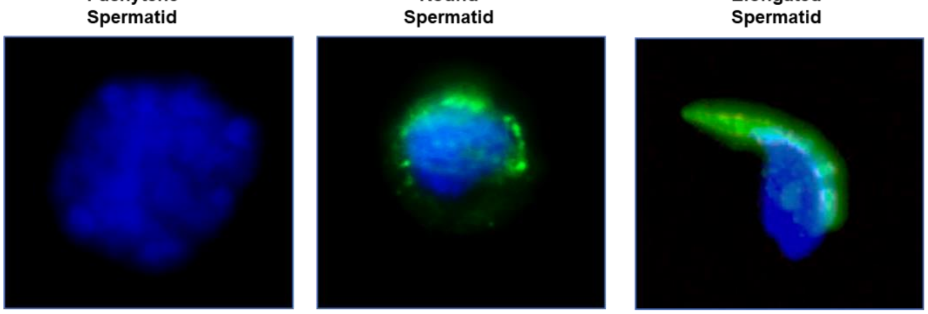

S2G

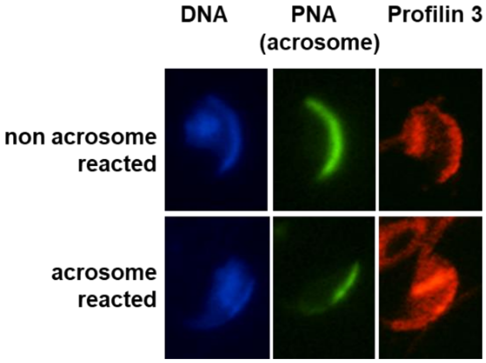

S2H

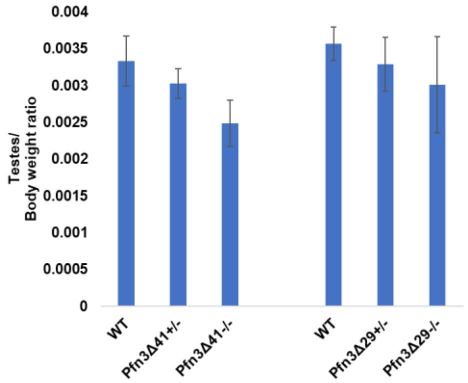

S2I

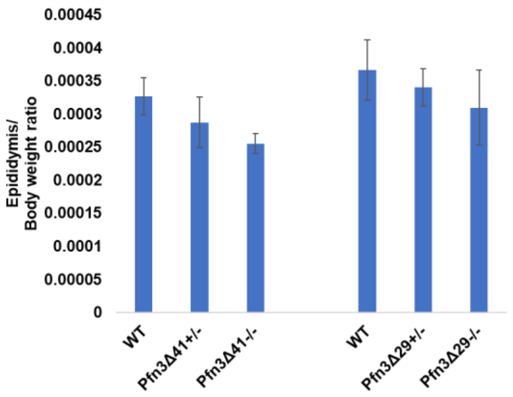

S2J

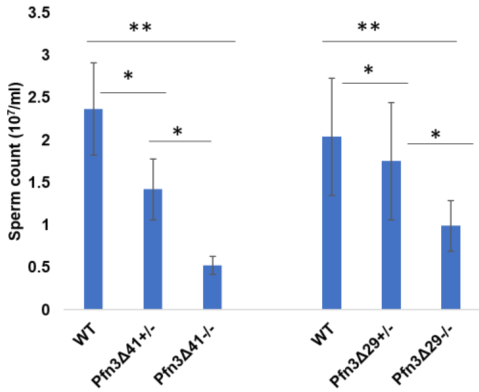

S2K

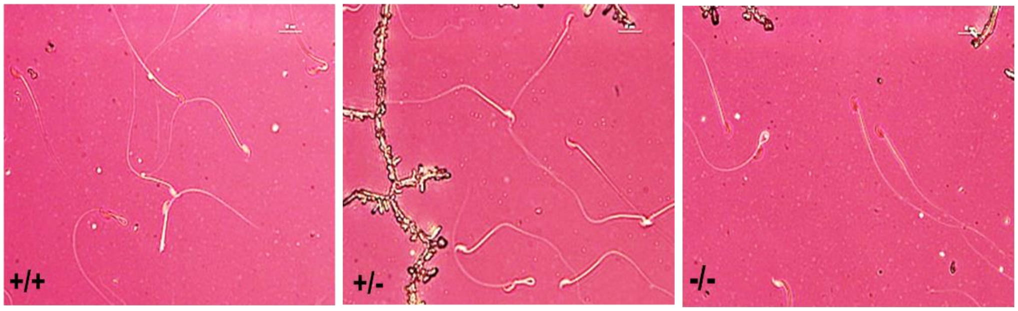

S2L

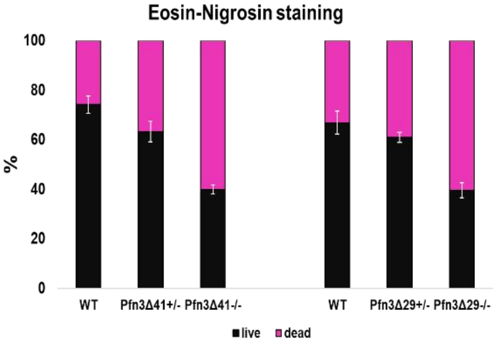

S2M

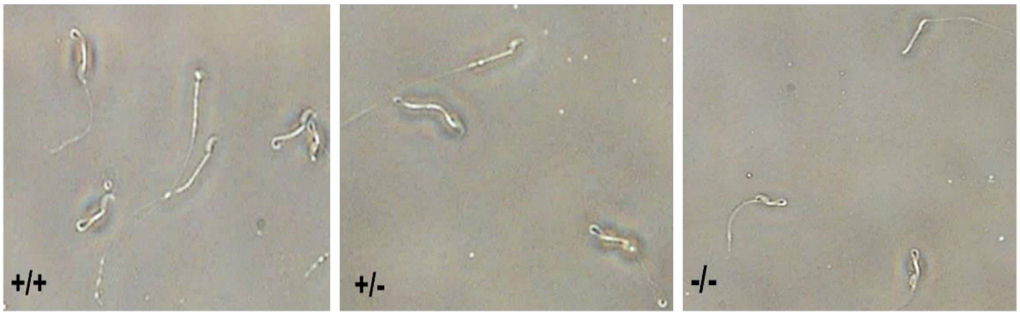

S2N

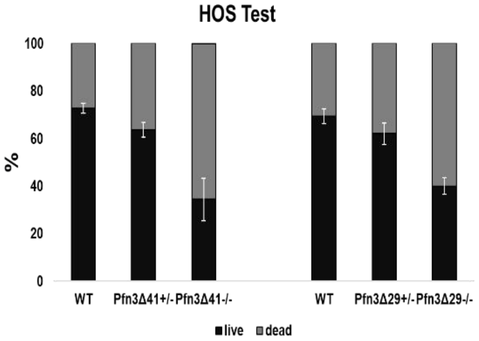

Supplementary Figure 2: **(A)** Genotyping for *Pfn3* deficient mice (P#1, 6, 9, 13, 14 and 16 are positive for the mutation). M= 100bp Plus ladder, 414bp PCR product represents WT allele, 160bp, 373bp and 385bp PCR products represent mutant allele**. (B)** Sequencing results using clustalW of *Pfn3*Δ254, *Pfn3*Δ41 and *Pfn3*Δ29. WT represents WT locus and mutation sequence represent with respective name. * indicates match base pairs. Dash line (-) represent deletion, and mismatch base pairs represent by no shape (nor * neither-). **(C)** Schematic of the established *Pfn3*-deficient alleles *Pfn3*Δ254, *Pfn3*Δ41 and *Pfn3*Δ29. Orange triangle marks the cleavage site result in frame shifts in the PFN3 reading frame leading to premature translational termination of the PFN3 protein. **(D)** Validation of *Pfn3* deficient mice. qRT-PCR was performed to check the relative expression of *Pfn3* mRNA in murine testis of wildtype, $Pfn3^{+/-}$ and $Pfn3^{-/-}$ mice for *Pfn3*Δ254, *Pfn3*Δ41 and *Pfn3*Δ29 mutation. Immunoblot against PFN3 protein following SDS gel electrophoresis of protein extracts from murine testis of $Pfn3^{+/+}$, $Pfn3^{+/-}$ and $Pfn3^{-/-}$ for each line. Beta-actin was used as control. Immunohistochemical staining of PFN3 on testicular sections of wildtype, $Pfn3^{+/-}$ and $Pfn3^{-/-}$ mice. Scale bar = 10µm. **(E)** IHC on brain, testis and kidney sections using PFN3 antibody. Scale bar = 100 µm. WB performed on various tissues isolated from mice using PFN3 antibody. B= Brain, H= heart, K=kidney, L=liver, M= muscle, P=pancreas, P28= post-natal day 28 testis, T=testis, O=ovary**. (F)** PFN3 staining in purified germ cells. Profilin 3 is not expressed in spermatocytes, is expressed broadly throughout the cytoplasm of round spermatids. In elongated spermatids, it is expressed along the entire exterior edge of the nucleus. **(G)** PFN3 expression in acrosome reacted and non-reacted spermatozoa. Nuclei were stained with Hoechst, acrosomes with FITC-PNA. Presence of FITC-PNA indicates the acrosome is intact, loss indicates the sperm underwent the acrosome reaction. In non-reacted sperm, PFN3 (red) is present in the acrosomal region. Staining remains strongly

in the head region (white arrow) after the acrosome is removed. **(H),(I)** Relative weight of testes and cauda epididymis are comparable between all three genotypes of *Pfn3*Δ41 and *Pfn3*Δ29 (n=13). **(J)** Sperm count comparison in $Pfn3^{+/+}$, $Pfn3^{+/-}$ and $Pfn3^{-/-}$ littermates (n=13). Scale bar = 10 µm. **(K)** Eosin and nigrosine staining of $Pfn3^{+/+}$, $Pfn3^{+/-}$ and $Pfn3^{-/-}$ sperms. Live= white sperm cells, pink=dead sperm cells**.** **(L)** Eosin and nigrosine staining on biological replicates (n=3) per genotype of $Pfn3^{+/+}$, $Pfn3^{+/-}$ and $Pfn3^{-/-}$ sperm. **(M)** Hypo-osmotic swelling test performed on $Pfn3^{+/+}$, $Pfn3^{+/-}$ and $Pfn3^{-/-}$ sperms. Tail curling= live sperm cells. Scale bar= 10µm. E&N staining and HOS test. **(N)** Hypo-osmotic swelling test on biological replicates (n=3) per genotype of $Pfn3^{+/+}$, $Pfn3^{+/-}$ and $Pfn3^{-/-}$ sperm. Scale bar = 10µm. At least 200 spermatozoa were evaluated per sample.

Supplementary Figure. 3

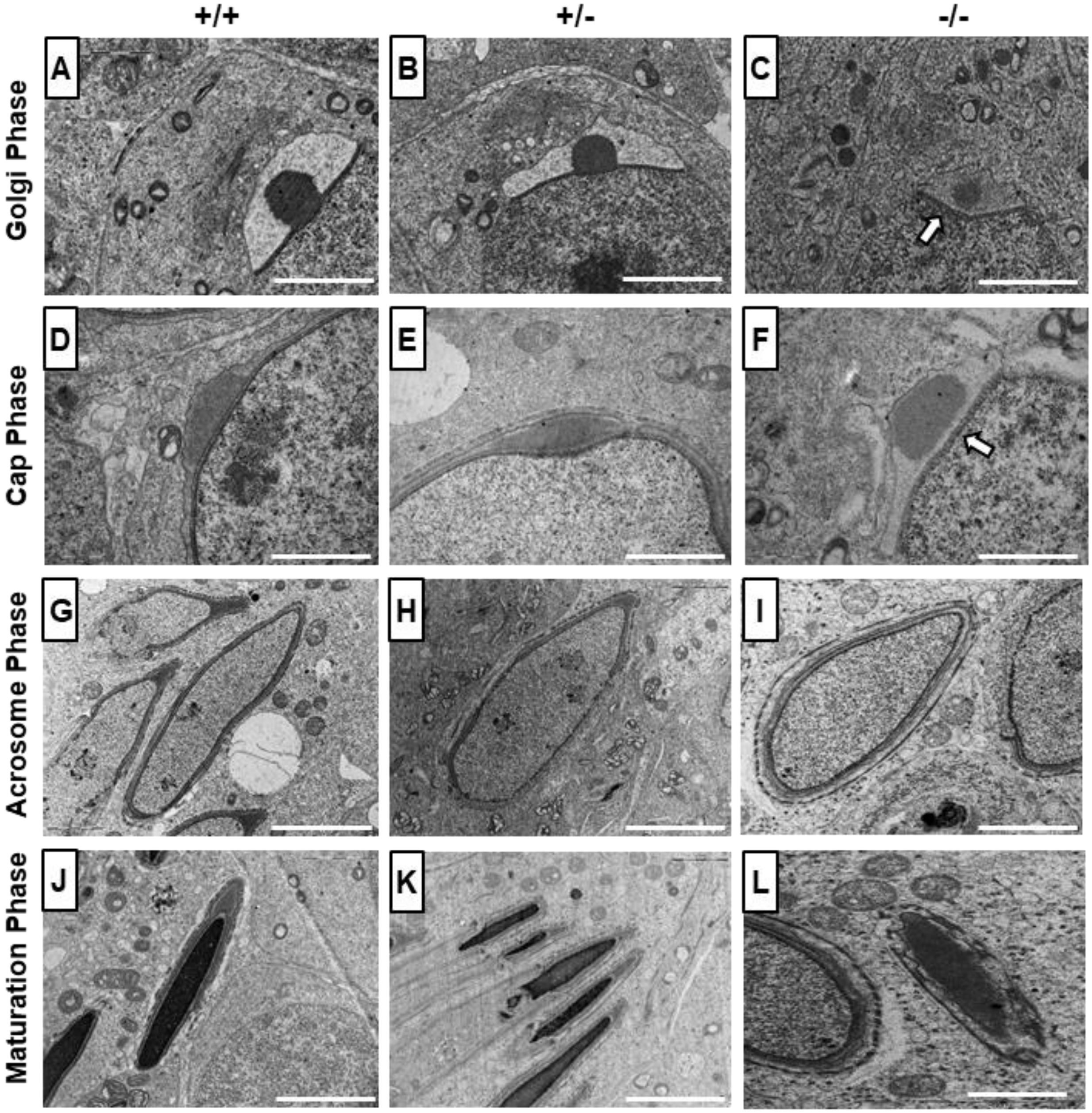

Supplementary Figure 3: TEM on developing spermatids in seminiferous tubules of *Pfn3*$^{+/+}$, *Pfn3*$^{+/-}$ and *Pfn3*$^{-/-}$ mice. In the Golgi phase, **(A)** *Pfn3*$^{+/+}$, **(B)** *Pfn3*$^{+/-}$ proacrosomal vesicles fused to form a strong acrosomal granule, **(C)** *Pfn3*$^{-/-}$ spermatids formed loosely clustered granular structure (white arrow). In the Cap phase, **(D)** *Pfn3*$^{+/+}$, **(E)** *Pfn3*$^{+/-}$ formed fine acrosomal caps, **(F)**

Pfn3$^{-/-}$ spermatids formed more bulgy and detached cap (white arrow). In the Acrosome phase, **(G)** *Pfn3*$^{+/+}$, **(H)** *Pfn3*$^{+/-}$ acrosome development as an arrow like covering, **(I)** *Pfn3*$^{-/-}$ spermatozoa showed malformed developing acrosome covering. In the Maturation phase, **(J)** *Pfn3*$^{+/+}$, **(K)** *Pfn3*$^{+/-}$ showed fully developed acrosome in elongated spermatozoa, **(L)** *Pfn3*$^{-/-}$ mature spermatozoa exhibit irregular acrosomal structure. Transmission electron micrographs showed malformed acrosome in *Pfn3* deficient sperm.

‘

Supplementary Figure. 4

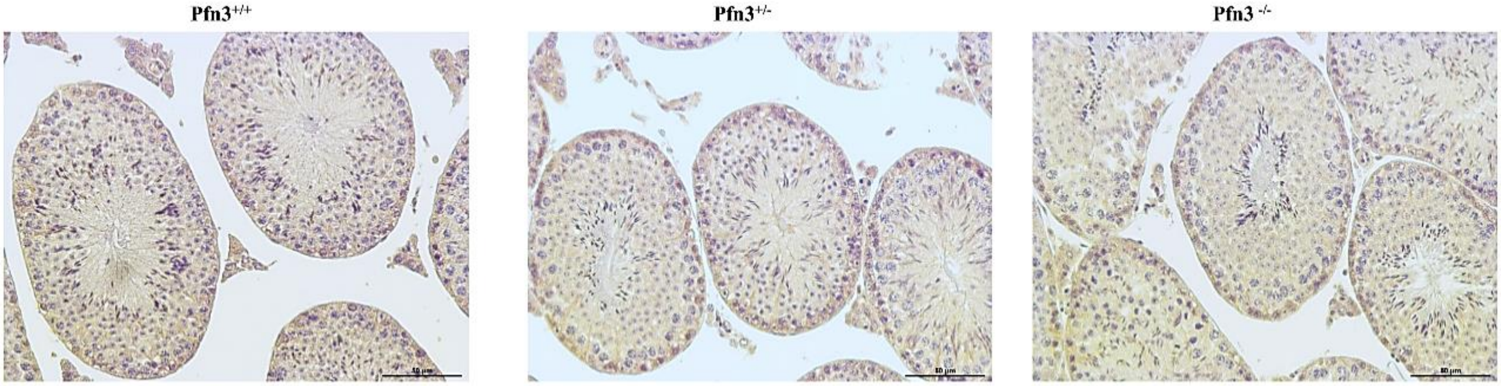

Supplementary Figure 4: IHC was performed using anti-Rab5 antibody on *Pfn3*$^{+/+}$, *Pfn3*$^{+/-}$ and *Pfn3*$^{-/-}$ testes section. Scale bar = 50 μm.

Supplementary Figure. 5

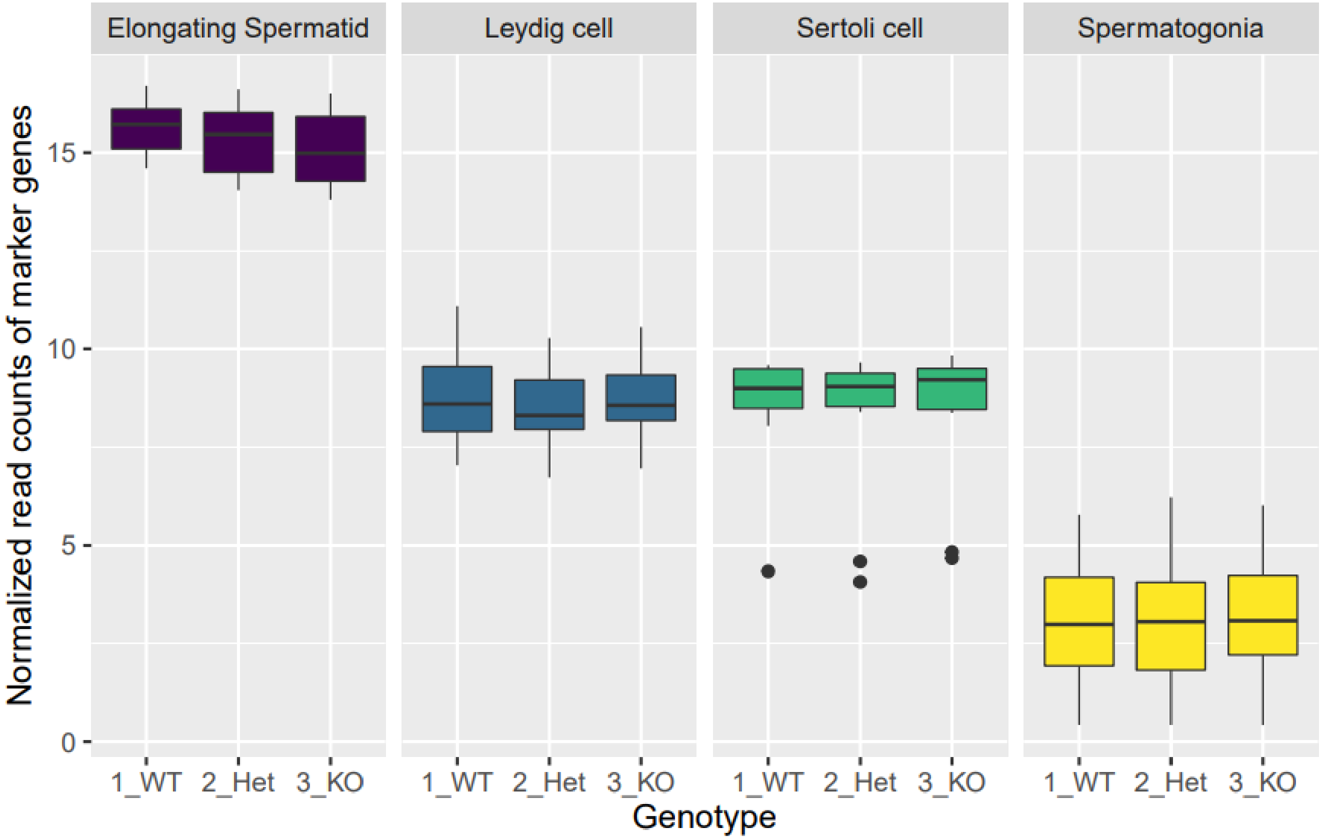

Supplementary Figure 5: Box plot of normalized read counts of marker genes in different cell types for all three genotypes. In elongating spermatozoa transition nuclear proteins and protamine's were used as a marker. Markers indicative for Leydig cells, Hsd17b3, Fabp3, Star, Insl3, Cyp11a1 and Cyp17a1, Sertoli cells Amhr2, Aard, Defb36 and Cst12 were used as markers, spermatogonia, Zbtb16, Plzf, Gfra1, Nanos3 and Lin28a were used.

Supplementary Figure. 6

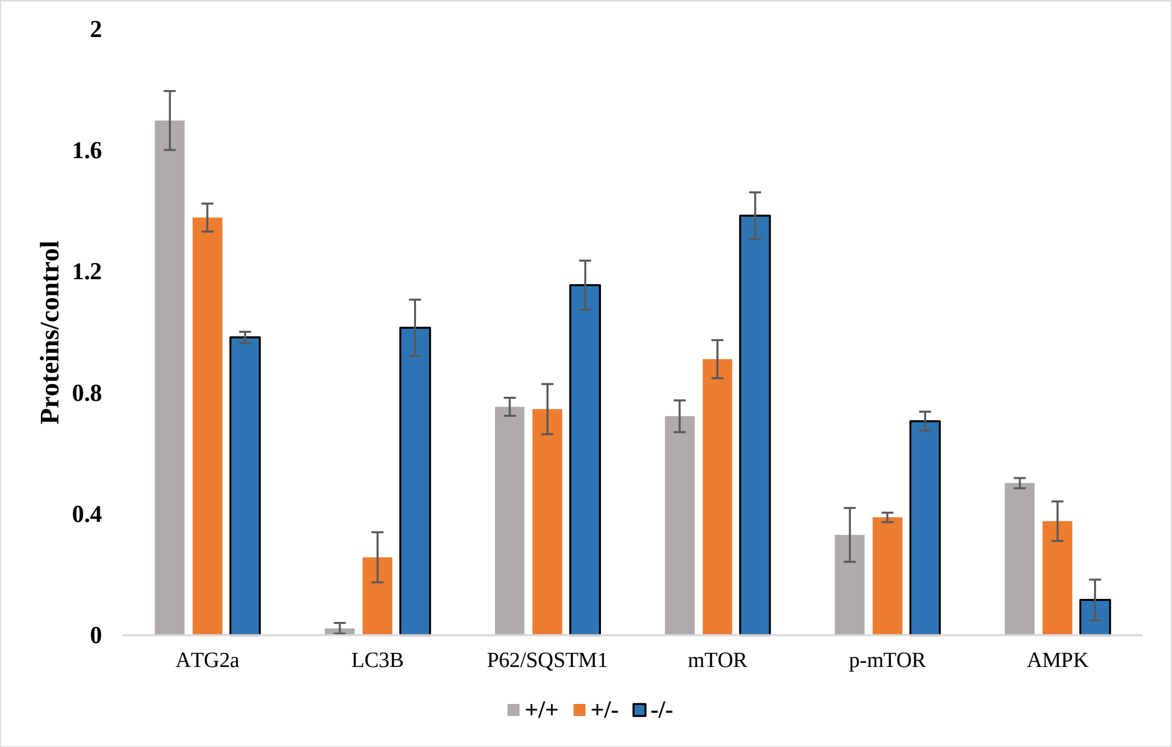

Supplementary Figure 6: Quantification of protein levels in all three genotypes. ATG2a and AMPK relatively quantified showing decreased protein levels in *Pfn3* deficient mice compared to controls. Similarly, LC3B, P62/SQSTM1, mTOR and p-mTOR protein levels are increased in *Pfn3* deficient mice indicating inhibition in autophagy. Details are present in main result part (Fig. 7).

μm.

Supplementary Figure. 7

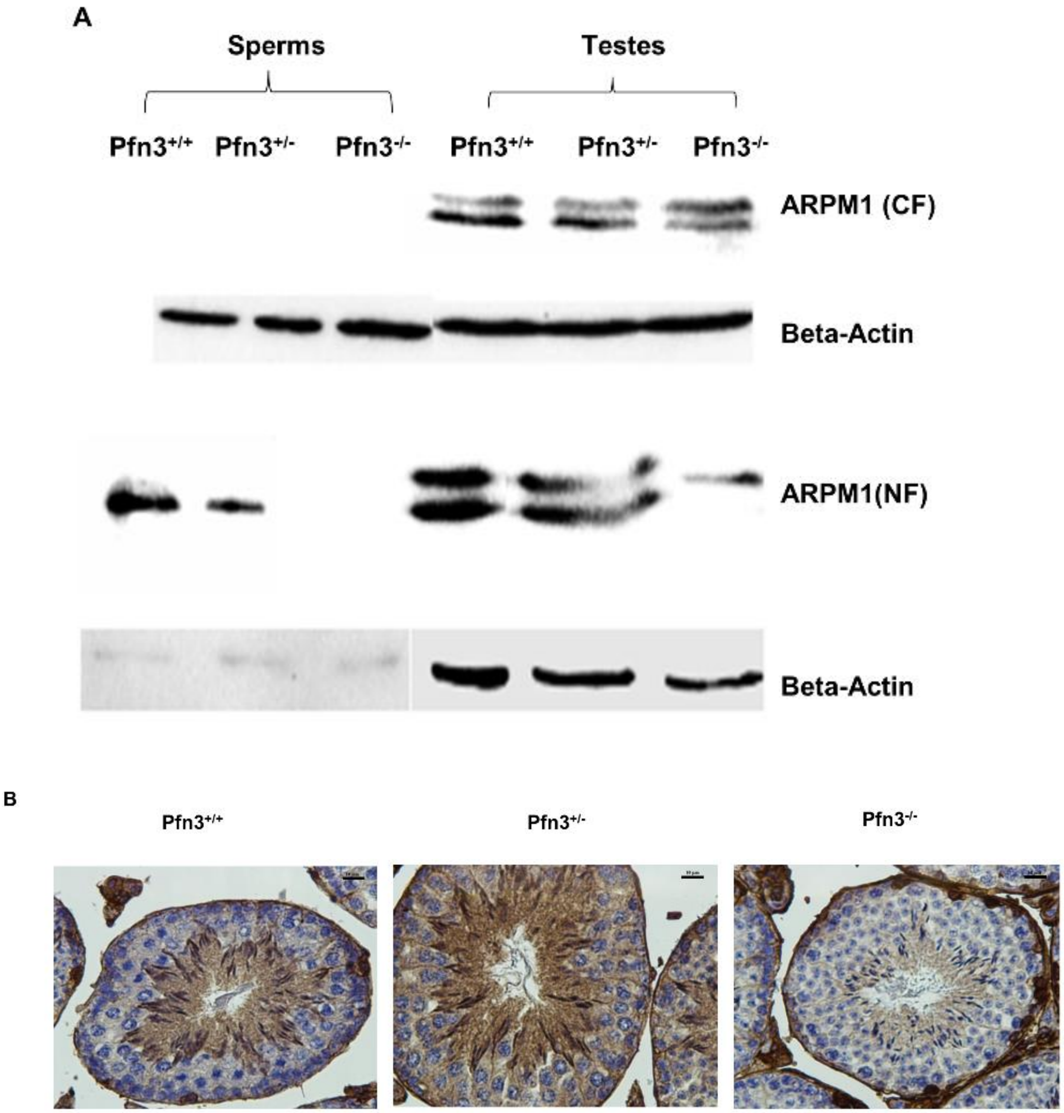

Supplementary Figure 7: Western blot analysis and IHC (immunohistochemical analysis) was performed using anti-ARPM1 antibody. **(A)** Western blot analysis of cytoplasmic and nuclear fraction from mice of *Pfn3*$^{+/+}$, *Pfn3*$^{+/-}$ and *Pfn3*$^{-/-}$ sperm and testes. **(B)** Immunohistochemical analysis using anti-ARPM1 antibody on *Pfn3*$^{+/+}$, *Pfn3*$^{+/-}$ and *Pfn3*$^{-/-}$ testes sections. Scale bar = 10 μm.

Supplementary Figure. 8

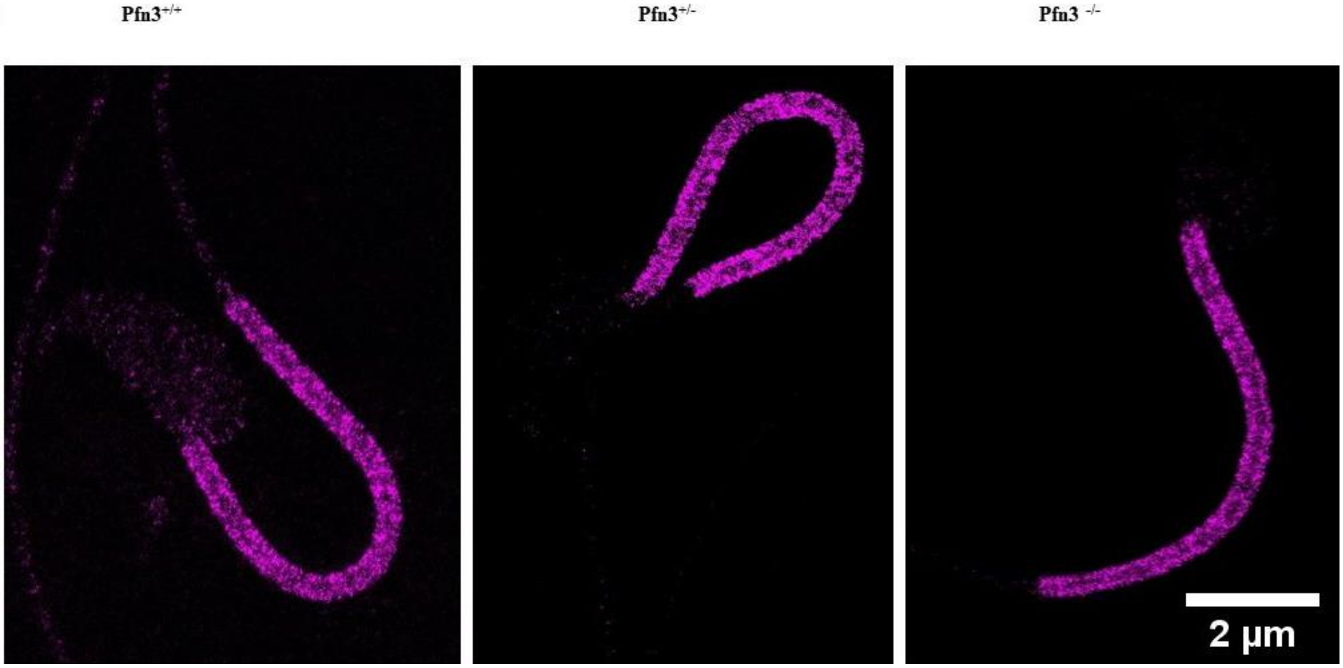

Supplementary Figure 8: Stimulated emission depletion (STED) microscopy was performed on mature sperm flagellum of *Pfn3*$^{+/+}$, *Pfn3*$^{+/-}$ and *Pfn3*$^{-/-}$ mice to detect the actin polymerization by using Phalloidine Atto-647 fluorescence. Scale bar = 2µm.

Supplementary Table 1: gRNAs and primers used in this study.

**gRNA Sequences**	**Top Strand oligo**	**Bottom strand oligo**
**Pfn3-gRNA1**	CACCGGTGCAGTGCTGCGGGATCAG	AAACCTGATCCCGCAGCACTGCACC
**Pfn3-gRNA2**	CACCGGACGGGCGTGCAATCTGTGT	CACCGGACGGGCGTGCAATCTGTGT
**In-Vitro transcribed Oligos**	**Forward primer**	**Reverse primer**
**T7-Pfn3-gRNA1**	TTAATACGACTCACTATAGGGTGCAGTGCTGCGGGATCAG	
**T7-Pfn3-gRNA1**	TTAATACGACTCACTATAGGGACGGGCGTGCAATCTGTGT	
**T7-sgRNA**		AAAAGCACCGACTCGGTGCCGCTGAGCCCAGAGCGCGTAG
**Genotyping PCR Primers**		
**Pfn3**	AGTGACTGGAAGGGCTACATCAGT	AGCACTGCTCACGCAGCCCACCAA
**qrt-PCR primers**		
**Pfn3-qrtPCR**	TGACTGAGCGGAGATGAGTG	TAGTCACGGATAACGCAGCA
**beta-Actin**	TGTTACCAACTGGGACGACA	GGGGTGTTGAAGGTCTCAAA
